# Differences in carbon sequestration capacity, rhizosphere microorganisms and metabolic functions among different herbaceous plants

**DOI:** 10.3389/fpls.2026.1849153

**Published:** 2026-06-17

**Authors:** Yu Zhou, Pushun Bian, Chen Yang, Jiayan Qu, Huaying Wang, Wei Gao

**Affiliations:** 1 College of Landscape Architecture, Changchun University, Changchun, Jilin, China; 2 School of Life Sciences, Northeast Normal University, Changchun, Jilin, China

**Keywords:** carbon sequestration, garden herbaceous plants, metabolic functions, metagenomics, rhizosphere microorganisms

## Abstract

Mitigating the rapid increase in global CO₂ concentrations necessitates a deeper understanding of plant-microbe symbiotic carbon sequestration. While previous research has predominantly focused on woody plants, the carbon sequestration potential and mechanisms of herbaceous plants and their rhizosphere microbiomes remain largely underexplored. To address this gap, this study employed metagenomic technology to systematically investigate the carbon sequestration capacities and metabolic mechanisms of seven plant species and their rhizosphere soil microorganisms. Plant physiological measurements were integrated with microbial functional profiles predicted via PICRUSt2. The results show that the rhizosphere soil microbial communities generally possess functional genes for carbon decomposition and carbon fixation, providing evidence for the coupling of intracellular decomposition and synthesis metabolism in microorganisms. Notably, Spearman correlation analysis established a direct statistical link between plant physiological performance and specific microbial metabolic pathways. These findings demonstrate a functional coupling between plant physiology and rhizosphere microbial carbon metabolism. By linking plant phenotypes to microbial gene pathways, this study reveals that herbaceous plants and their rhizosphere microbiomes form an integrated carbon sequestration system. Therefore, leveraging such plant-soil interactions offers a promising strategy to enhance ecosystem carbon sinks and mitigate rising atmospheric CO₂.

## 1 Introduction

The world is currently grappling with unprecedented climate change, predominantly driven by global warming. Since the Industrial Revolution, human activities, such as fossil fuel combustion and land use changes, have led to a rapid surge in atmospheric concentrations of greenhouse gases, including carbon dioxide (CO_2_) and methane (CH_4_). Assessment reports released by the Intergovernmental Panel on Climate Change (IPCC) clearly demonstrate that this anthropogenically amplified greenhouse effect has triggered a sharp rise in the average global temperature, with the global surface temperature having increased by 1.09 °C between 2010 and 2020. This warming trend has precipitated a series of severe global environmental crises, including melting polar glaciers, rising sea levels, and the frequent occurrence of extreme weather events (R et al., 2025). Addressing climate change and mitigating global warming has thus become one of the most urgent challenges confronting the international community. In response to global warming, China has explicitly put forward its “dual carbon” goals, with overarching targets of reaching a carbon peak before 2030 and achieving carbon neutrality before 2060 (Xueqing et al., 2023).

From the perspective of Earth system science, terrestrial ecosystems play a pivotal role in regulating the atmospheric carbon balance. Among these ecosystems, soil serves as the largest organic carbon reservoir on land, with an estimated global soil organic carbon storage of approximately 2500 Pg (Rattan et al., 2021), nearly three times the size of the atmospheric carbon pool (approximately 880 Pg C). Even subtle dynamic changes in soil carbon can exert profound impacts on the global carbon balance and climate change. Soil organic carbon (SOC) is not a homogeneous substance but comprises multiple components with distinct turnover rates and stability. These components mainly include: dissolved organic carbon (DOC); microbial biomass carbon (MBC), which is closely associated with microbial activity; particulate organic carbon (POC); and mineral associated organic carbon (MAOC), a chemically stable fraction bound to mineral particles. The proportions of these components directly determine the stability of the soil carbon pool and its potential for carbon sequestration.

Rhizosphere soil is strongly influenced by plant root activities and serves as a critical hotspot for soil carbon input, transformation, and storage (Wang et al., 2025). Plants can allocate up to 20%–40% of photosynthetically fixed carbon to the rhizosphere through root exudates and litter inputs. Due to their unique developmental backgrounds, metabolic pathways, and secondary metabolites, different plant species secrete root exudates with distinct chemical compositions. By releasing exudates (such as organic acids, phenolics, and flavonoids), plant roots can alter the rhizosphere microenvironment, thereby screening surrounding microorganisms and assembling rhizosphere microbial communities adapted to their own physiological and ecological strategies (Yian et al., 2016). However, a large body of current research has been primarily focused on trees, shrubs (Zongrui et al., 2023), and woody lianas (Han, 2005; Liu et al., 2016), while insufficient attention has been paid to ornamental herbaceous plants widely used in landscaping and ecological restoration. These plants often possess more abundant secondary metabolites (such as terpenoids, phenolic acids, and alkaloids), which may exert stronger selective pressure on microorganisms, thereby forming functionally specific rhizosphere effects.

In this study, seven common greening herbaceous plants in Changchun were investigated during the peak growth period in August.: *Hosta ensata* F. Maek. (HE), *Pseudolysimachion spicatum* (L.) Opiz (PS), *Rudbeckia hirta* L. (RH), *Gaillardia pulchella* Foug. (GP), *Salvia jponica* Thunb. (SJ), *Lycopus lucidus* Turcz. ex Benth. (LL), and *Hemerocallis fulva* ‘Golden Doll’ (HF). This study aimed to investigate: the carbon sequestration capacity across these species; the diversity within the rhizosphere bacterial and fungal community structures; and identify plants exhibiting high carbon sequestration alongside rhizosphere microorganisms abundant in beneficial bacteria.

## 2 Materials and methods

### 2.1 Study site

Experimental field of the College of Landscape Architecture, Changchun University, Changchun, Jilin Province, China (43.83°N, 125.30°E), with an annual average temperature of 4.8 °C, maximum temperature of 39.5 °C, minimum temperature of -39.8 °C, and sunshine duration of 2688 hours. According to the classification of the Resource and Environment Science and Data Center, Chinese Academy of Sciences (https://www.resdc.cn), the soil at the experimental site was classified as meadow black soil with a clay loam texture and was slightly alkaline. The seven plant species were planted as monocultures in the experimental plots and allowed to grow under natural field conditions.

### 2.2 Measurement and calculation of plant photosynthetic physiological indices

The photosynthetic rate of individual plants was measured using a LI-6800 portable photosynthesis system under natural light conditions, with measurements taken every 2 hours from 8:00 to 16:00. Three healthy plants with similar growth statuses were selected for each species, and three similar leaves with comparable sizes and appearances were chosen for measurement. The average value was calculated. Canopy photographs were taken using a canopy analyzer, and clear photos that easily distinguished the sky and canopy were analyzed using Gap Light Analyzer (GLA) version 2.0 to obtain the leaf area index (LAI). Based on the principle of daily assimilation calculation by Han Huanjin, plant carbon sequestration was estimated using the measured net assimilation of plants on the measurement day (Yanting et al., 2019).


$$\text{P}=\sum_{i=1}^{n}\frac{\left(P_{\left(i+1\right)}+P_{i})\times (t_{\left(i+1\right)}-t\right)}{2\times 1000}\times 3600$$

$$q=\frac{\sum_{i=1}^{n}P_{i}}{n}$$

$${\text{wCO}}_{2}\frac{P\times 44}{1000}\times (1-20\%)$$

$${\text{WCO}}_{2}={\text{wCO}}_{2}\times \text{LAI}$$

P represents the daily total net assimilation per unit leaf area; P_i_ is the instantaneous photosynthetic utilization rate at the initial measurement point; P_i+1_ is the instantaneous photosynthetic utilization rate at the i+1 measurement point; t is the instantaneous time (in hours) at the initial measurement point; ti+1 is the instantaneous time at the i+1 measurement point; n is the number of measurements; q is the daily average photosynthetic rate; 3600 represents 3600 seconds per hour; and 1000 represents that 1 mmol corresponds to 1000 μmol. The daily net carbon sink per unit leaf area is denoted as wCO_2_. The nighttime respiration consumption of the tested plant was calculated as 20% of the total daily net assimilation of that species (Zhang et al., 2013). 44 is the molar mass of CO_2_. The daily net carbon sequestration per unit land area of this plant is represented by WCO_2_, and LAI is the leaf area index.

### 2.3 Soil sampling and chemical property determination

Researchers carefully selected seven types of vigorous, healthy, and completely disease-free herbaceous plants from the experimental fields of the College of Landscape Architecture, Changchun University, and measured the photosynthetic capacity of three individual plants per type. For each plant, a five-point sampling method was employed to collect rhizosphere soil from a depth of 0–10 cm within 2 cm radius around the plant roots. After removing loosely attached soil, the rhizosphere soil closely adhering to the root system was collected. The rhizosphere soil from the three individuals of each plant type was pooled and divided into two portions: one portion was stored at 4 °C for determination of soil carbon components, and the other was stored at -80 °C for subsequent DNA extraction and chemical analysis. During measurement, each indicator was measured four times to minimize error.

SOC was determined using the potassium dichromate oxidation method (Qinglin et al., 2025). DOC was determined by water extraction followed by the potassium dichromate oxidation method as described by Ciavatta et al. (1991). POC and MAOC were separated and determined according to the method described by Six and Paustian (Six et al., 2000). According to relevant methods described in Soil Agricultural Chemistry Analysis. Soil pH was determined using the potentiometric method (soil:water ratio of 1.0:2.5) (Akira et al., 2023), total nitrogen (TN) content was measured by the Kjeldahl method, total phosphorus (TP) content was determined by NaOH fusion molybdenum antimony resistance spectrophotometry, and total potassium (TK) was measured by flame photometry.

### 2.4 DNA extraction, sequencing and data processing

Total microbial genomic DNA was extracted from samples using the E.Z.N.A.^®^ soil DNA Kit (Omega Bio-tek, Norcross, GA, U.S.) according to manufacturer’s instructions. The quality and concentration of DNA were determined by 1.0% agarose gel electrophoresis and a NanoDrop 2000 spectrophotometer (Thermo Scientific, United States). The DNA was kept at -80 °C prior to further use. The hypervariable regions V3-V4 of the bacterial 16S rRNA gene were amplified with primer pairs 338F (5’-ACTCCTACGGGAGGCAGCAG-3’) (Liu et al., 2016) and 806R(5’-GGACTACHVGGGTWTCTAAT-3’) (Chen et al., 2018) by T100 Thermal Cycler PCR thermocycler (BIO-RAD, USA). The PCR reaction mixture included 4 μL 5 × Fast Pfu buffer, 2 μL 2.5 mM dNTPs, 0.8 μL each primer (5 μM), 0.4 μL Fast Pfu polymerase, 10 ng of template DNA, and ddH_2_O to a final volume of 20 µL. PCR amplification cycling conditions were as follows: initial denaturation at 95 °C for 3 min, followed by 27 cycles of denaturing at 95 °C for 30 s, annealing at 55 °C for 30 s, extension at 72 °C for 45 s, and single extension at 72 °C for 10 min, ending at 4 °C. The PCR product was extracted from 2% agarose gel, purified using the PCR Clean-Up Kit (YuHua, Shanghai, China) according to the manufacturer’s instructions and quantified using the Qubit 4.0 (Thermo Fisher Scientific, USA).

Purified amplicons were pooled in equimolar amounts and paired-end sequenced on an Illumina Nextseq2000 platform (Illumina, San Diego, USA) according to the standard protocols by Majorbio Bio-Pharm Technology Co. Ltd. (Shanghai, China).

#### 2.4.1 Illumina sequencing and data processing

Equimolarly pooled amplicons underwent paired-end sequencing on an Illumina NextSeq 2000 system (Illumina, San Diego, CA, USA) at Majorbio Bio-Pharm Technology Co., Ltd. (Shanghai, China). Raw FASTQ files were demultiplexed and subjected to quality control using fastp v0.19.6 [2]. Paired reads were subsequently merged using FLASH v1.2.7 (Magoč and Salzberg, 2011).

High-quality sequences were clustered into Operational Taxonomic Units (OTUs) at 98.65% identity via UPARSE 7.1 (Kim et al., 2014; Edgar, 2013). After removing chloroplast sequences manually, the OTU table was rarefied to 20,000 reads per sample to normalize sequencing depth, yielding an average Good’s coverage of 99.09%. Taxonomic annotation of representative sequences (the most abundant sequence per OTU) was performed against the Silva v138 database using RDP Classifier v2.14 (Wang and Cole, 2024) at a 0.7 confidence threshold. Finally, microbial metagenomic functions were predicted based on these representative sequences utilizing the standard PICRUSt2 pipeline (Douglas et al., 2020).

#### 2.4.2 Statistical analysis

Bioinformatic analysis of the soil was carried out using the Majorbio Cloud platform (https://cloud.majorbio.com). Based on the OTUs information, rarefaction curves and alpha diversity indices including observed OTUs, Chao1 richness, Shannon index and Good’s coverage were calculated with Mothur v1.30.1 (Schloss et al., 2009). The similarity among the microbial communities in different samples was determined by principal coordinate analysis (PCoA) based on Bray-curtis dissimilarity using the vegan v2.5–3 package. The PERMANOVA test was used to assess the percentage of variation explained by the treatment along with its statistical significance using vegan v2.5–3 package. The linear discriminant analysis (LDA) effect size (LEfSe) (Segata et al., 2011) (http://huttenhower.sph.harvard.edu/LEfSe) was performed to identify the significantly abundant taxa (phylum to genera) of bacteria among the different groups (Note: When a sequence cannot be annotated to a known genus at the genus level, it is uniformly marked as unclassified_[family name]). The distance based redundancy analysis (db-RDA) was performed using the vegan v2.5–3 package to investigate the effect of soil physicochemical properties on soil bacterial community structure. Qiime (2020.2.0) Beta diversity distance matrices were calculated, and NMDS analysis and plotting were performed using the vegan package (version 2.4.3) in R language (version 3.3.1).

**Figure 1:**
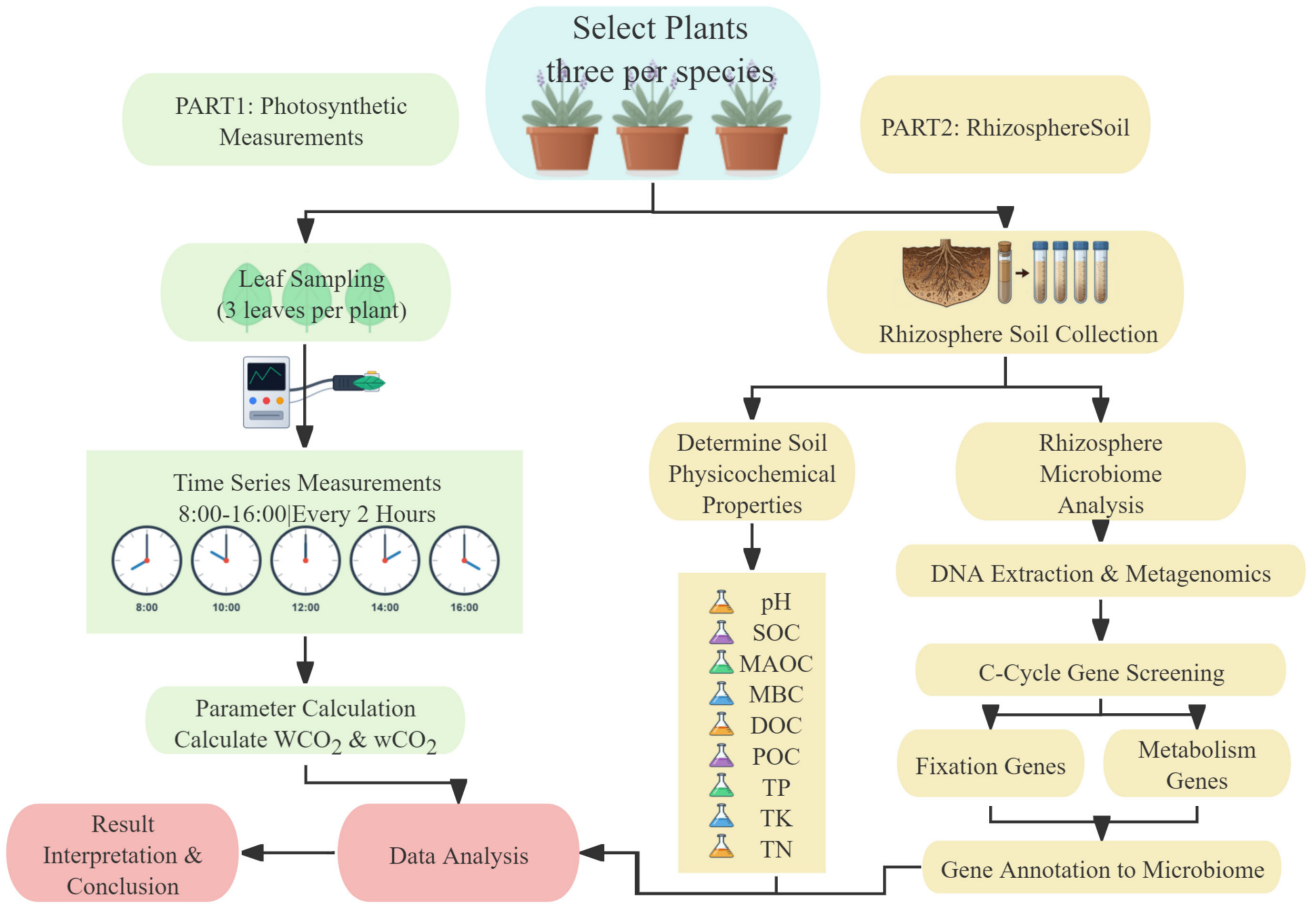
Flowchart of the experiment.

The study consists of two main parts: photosynthetic measurements and rhizosphere soil analysis. For photosynthetic measurements, three leaves per plant were sampled from three plants per species, and measurements were taken every two hours from 8:00 to 16:00. For rhizosphere soil analysis, collected soil was used to determine physicochemical properties (pH, SOC, MAOC, MBC, DOC, POC, TP, TK, and TN) and for metagenomic analysis following DNA extraction. Parameters WCO_2_ and wCO_2_ were calculated, and together with gene annotation results from the rhizosphere microbiome, data analysis was performed to reach the final interpretation and conclusion (**Figure 1**).

## 3 Results and analysis

### 3.1 Comparison of carbon sequestration

**Figure 2:**
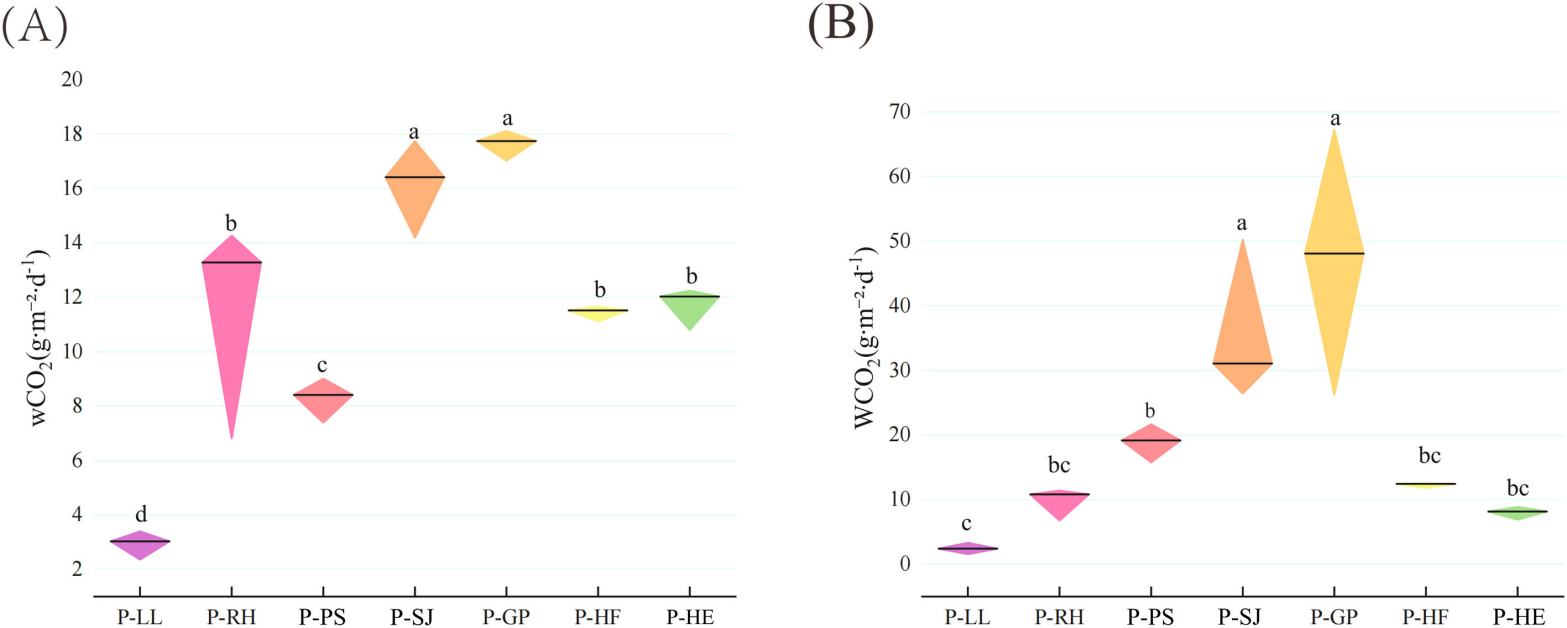
Carbon sequestration among different plants. **(A)** for wCO_2_ and **(B)** for WCO_2_. Different letters indicate significant differences between them.

To compare the carbon sequestration capacity of different plants, the daily CO_2_ fixation per unit leaf area (wCO_2_) and daily CO_2_ fixation per unit land area (WCO_2_) of seven herbaceous species were measured. The results showed that, both wCO_2_ and WCO_2_ varied substantially among species. *Gaillardia pulchella* Foug. (P-GP) and *Salvia japonica* Thunb. (P-SJ) exhibited significantly higher values than the other five species (p < 0.05). (For brevity, combined abbreviations such as P-GP, S-GP, and RS-GP are used in this paper. The prefixes “P-”, “S-”, and “RS-” are defined as follows: P- denotes the corresponding plant, S- denotes rhizosphere microorganisms, and RS- denotes rhizosphere soil. The suffixes represent the seven plant species (species abbreviations have been defined at their first appearance in the text). Accordingly, P-GP represents plant-*Gaillardia pulchella* Foug., S-GP represents rhizosphere microorganism-*Gaillardia pulchella* Foug., and RS-GP represents rhizosphere soil-*Gaillardia pulchella* Foug.; the same pattern applies to the remaining species.) (**Supplementary Table 13**). Specifically, P-GP had mean wCO_2_ of 17.63 and mean WCO_2_ of 47.18, which were 5.50 and 17.73 times higher, respectively, than the lowest values in *Lycopus lucidus* Turcz. ex Benth. (P-LL). Similarly, P-SJ had mean values of wCO_2_ of 16.12 and mean values of WCO_2_ of 35.92, corresponding to 6.02 fold and 17.35 fold increases relative to P-LL. These results indicate that *Gaillardia pulchella* Foug and *Salvia japonica* Thunb possess markedly higher carbon sequestration capacity than *Lycopus lucidus* Turcz. ex Benth under the same growing conditions (**Figure 2**).

**Table 1:** Differences in rhizosphere soil chemical properties among different plants.

	SOC (g/kg)	MAOC (g/kg)	MBC (mg/kg)	DOC (mg/kg)	POC (g/kg)	pH	TN	TP	TK
RS-LL	26.158 ± 7.033	22.878 ± 7.069	620.228 ± 0.068 ^c^	418.317 ± 1.412 ^e^	3.28 ± 0.036 ^c^	7.33 ± 0.039 ^c^	1.223 ± 0.005 ^a^	0.614 ± 0.086 ^a^	0.737 ± 0.175 ^c^
RS-RH	23.958 ± 1.867	21.791 ± 1.866	357.613 ± 0.153 ^f^	271.189 ± 2.801 ^f^	2.167 ± 0.012 ^f^	7.43 ± 0.008 ^b^	0.838 ± 0.005 ^c^	0.421 ± 0.059 ^d^	0.505 ± 0.118 ^c^
RS-PS	34.298 ± 1.732	30.623 ± 1.753	655.57 ± 1.431 ^b^	464.159 ± 1.699 ^b^	3.675 ± 0.071 ^b^	7.42 ± 0.008 ^b^	0.748 ± 0.005 ^e^	0.376 ± 0.053 ^b^	0.451 ± 0.105 ^b^
RS-SJ	32.758 ± 6.009	28.894 ± 6.009	707.87 ± 1.154 ^a^	481.921 ± 0.847 ^a^	3.864 ± 0.021 ^a^	7.37 ± 0.014 ^c^	0.71 ± 0 ^f^	0.355 ± 0.050 ^e^	0.429 ± 0.104 ^d^
RS-GP	27.698 ± 14.761	24.116 ± 14.799	528.368 ± 0.148 ^e^	453.194 ± 1.825 ^c^	3.582 ± 0.052 ^e^	7.34 ± 0.02 ^c^	0.98 ± 0 ^b^	0.49 ± 0.069 ^b^	0.591 ± 0.144 ^a^
RS-HF	30.338 ± 5.712	28.558 ± 5.676	312.833 ± 0.052 g	220.876 ± 1.296 g	1.78 ± 0.041 g	7.53 ± 0.037 ^a^	0.568 ± 0.005 g	0.286 ± 0.040 ^a^	0.342 ± 0.079 ^d^
RS-HE	36.058 ± 7.57	32.549 ± 7.564	650.91 ± 1.422 ^d^	435.274 ± 1.23 ^d^	3.509 ± 0.036 ^d^	7.51 ± 0.051 ^a^	0.813 ± 0.005 ^d^	0.409 ± 0.057 ^c^	0.49 ± 0.115 ^c^

SOC, soil organic carbon; MAOC, mineral-associated organic carbon; MBC, microbial biomass carbon; DOC, dissolved organic carbon; POC, particulate organic carbon; pH, soil pH value, TN, total nitrogen, TP, total phosphorus, TK, total potassium values are mean ± standard deviation; values with different lowercase letters indicate significant differences among rhizosphere soils of different plants (p < 0.05).

### 3.2 Differences in rhizosphere soil chemical properties among different plants

To evaluate the effects of different plant species on rhizosphere soil chemical properties, we measured seven indices: soil organic carbon (SOC), mineral-associated organic carbon (MAOC), microbial biomass carbon (MBC), dissolved organic carbon (DOC), particulate organic carbon (POC), pH, total nitrogen (TN), total phosphorus (TP), and total potassium (TK). One-way ANOVA was performed to compare differences among the seven plant species. The results showed that SOC and MAOC did not differ significantly among rhizosphere soils of the seven species (p > 0.05). In contrast, MBC, DOC, and POC exhibited significant interspecific differences (p < 0.05). Specifically, the highest values of MBC, DOC, and POC were all observed in the rhizosphere soil of *Salvia japonica* Thunb. (RS-SJ), followed by *Polygonum* sp. (RS-PS), while the lowest values were found in *Hemerocallis fulva* ‘Golden Doll’ (RS-HF). Soil pH differed significantly among species (p < 0.05), but the variation was small, with all values ranging from 7.33 to 7.53 (weakly alkaline). TN content was highest in the rhizosphere soil of *Lycopus lucidus* Turcz. ex Benth. (RS-LL) at 1.223 ± 0.005, which was 2.15 times that of RS-HF. TP content was also highest in RS-LL (0.614 ± 0.086), 0.328 g/kg higher than the lowest value in RS-HF. TK content was highest in RS-LL (0.737 ± 0.175), 2.11 times that of the lowest in RS-HF (**Table 1**).

### 3.3 Differences in rhizosphere microbial communities among different plants

**Figure 3:**
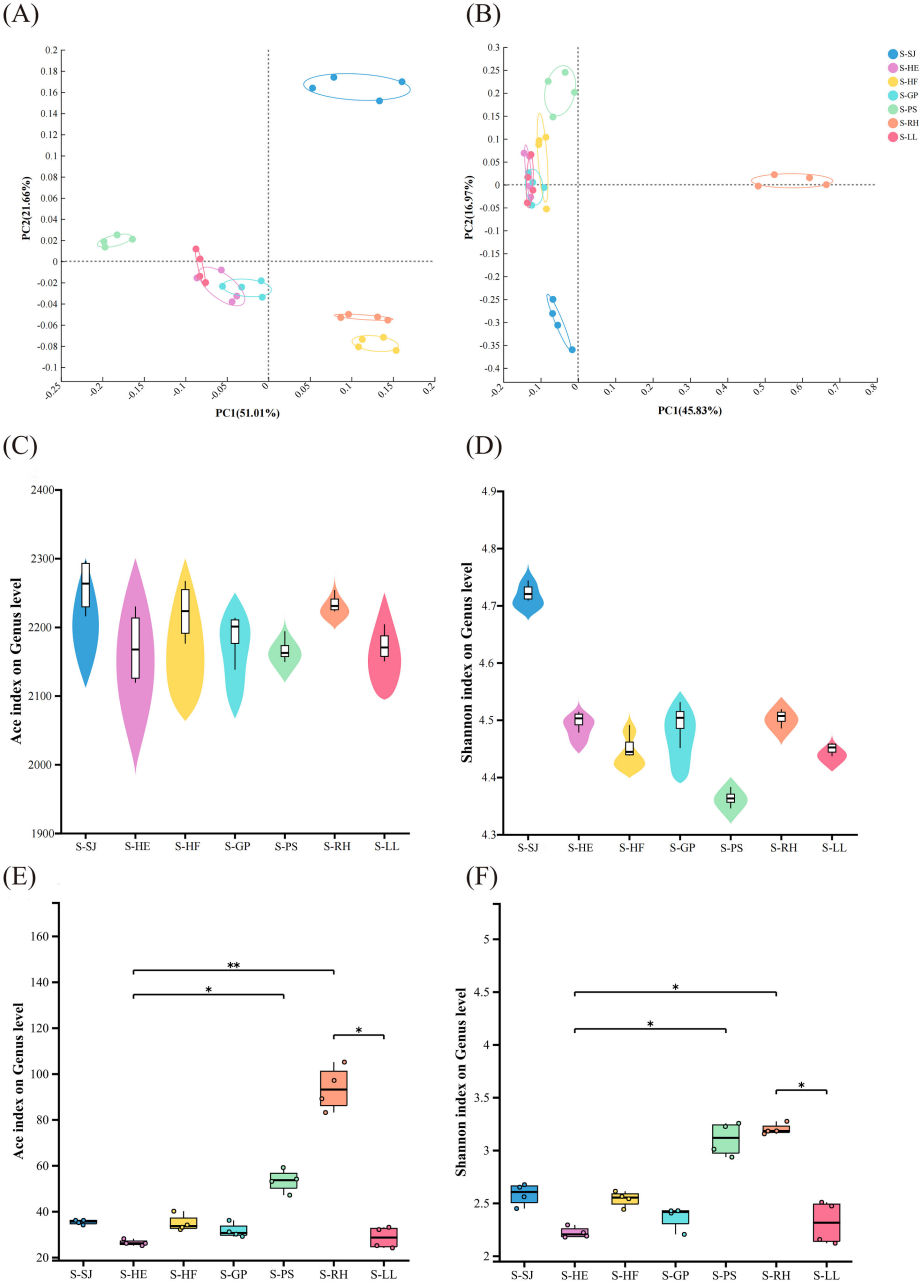
The differences of soil microorganisms in the rhizosphere of different plants. **(A)** Principal component analysis of bacterial communities, **(B)** Principal component analysis of fungal communities, **(C)** represents the bacterial ace index, **(D)** represents the bacterial Shannon index, **(E)** represents the fungal ace index, and **(F)** represents the fungal Shannon index. The symbols * and ** denote statistically significant differences between groups as follows: * p < 0.05, ** p < 0.01.

A total of 2,917 bacterial genera and 150 fungal genera were identified. Analysis of bacterial and fungal alpha and beta diversity demonstrated that the rhizosphere soils of different herbaceous plants significantly influenced microbial community diversity (p < 0.05) (**Figures 3A, B**). Specifically, regarding bacterial communities, sample S-SJ exhibited relatively high diversity and species richness, whereas S-PS presented relatively low values (**Figures 3C, D**). Conversely, an opposing trend was observed for fungal diversity and richness, where S-SJ showed relatively low levels and S-RH showed relatively high levels (p < 0.05) (**Figures 3E, F**).

Furthermore, beta diversity analysis indicated that microbial species composition varied substantially in S-SJ compared to the other groups, highlighting its distinct microbial structure. In contrast, the microbial communities associated with S-HE, S-GP, and S-LL displayed high compositional similarity.

**Figure 4:**
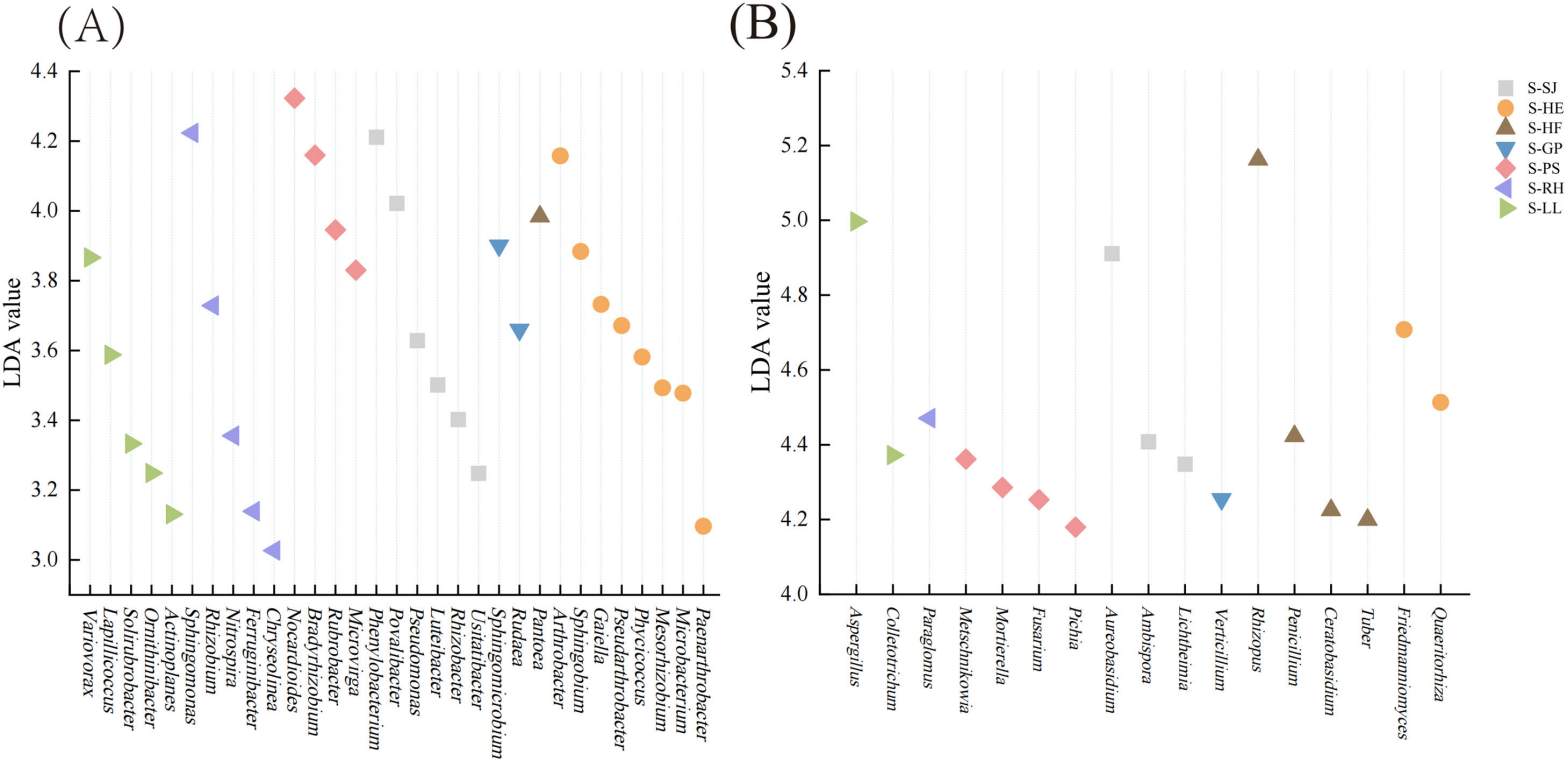
Distribution of bacterial **(A)** and fungal **(B)** genera in the rhizosphere soil of different plant species.

To identify specific microbial biomarkers for each plant species, we performed Linear discriminant analysis Effect Size (LEfSe) analysis on the seven groups. A total of 61 bacterial taxa with LDA scores > 3.0 were identified as biomarkers, with distinct distribution patterns across the seven groups (**Figure 4A**). In S-SJ, the bacterial community was dominated by the phylum Proteobacteria, particularly groups associated with the degradation of aromatic compounds and plant growth promotion; key taxa included *Phenylobacterium*, *Povalibacter*, *Pseudomonas*, and *Luteibacter*. In S-HE, the phylum Actinobacteria dominated, with key taxa including *Arthrobacter*, *Gaiella*, *Pseudarthrobacter*, *Phycicoccus*, *Microbacterium*, *Paenarthrobacter*, and *Sphingobium*. In S-HF, the only identified genus was *Pantoea*. In S-GP, only *Sp*hingomicrobium and Rudaea were identified, representing relatively few specific microbial groups. In S-PS, key taxa included Nocardioides, Rubrobacter, Bradyrhizobium, and Microvirga. In S-RH, the microbial community included groups involved in nitrogen and carbon cycles, such as Nitrospira, Rhizobium, Ferruginibacter, and Chryseolinea. In S-LL, key taxa included Solirubrobacter and Actinoplanes. For each group, additional biomarker taxa that could not be classified to the genus level are provided in the attached **Supplementary Table 3**.

After excluding non fungal groups, a total of 17 fungal taxa with LDA scores > 3.0 were identified across the seven plant groups (**Figure 4B**; **Supplementary Table 4**). In S-SJ, the fungal biomarkers were Lichtheimia, Ambispora, and Aureobasidium. S-RH had only Paraglomus. S-PS contained Metschnikowia, Pichia, Mortierella, and Fusarium. S-LL had Colletotrichum and Aspergillus. S-HE included Quaeritorhiza and Friedmanniomyces. S-HF had four biomarkers, Tuber, Rhizopus, Ceratobasidium, and Penicillium. S-GP had only Verticillium.

### 3.4 Carbon cycling genes in rhizosphere soils of different plants

**Figure 5:**
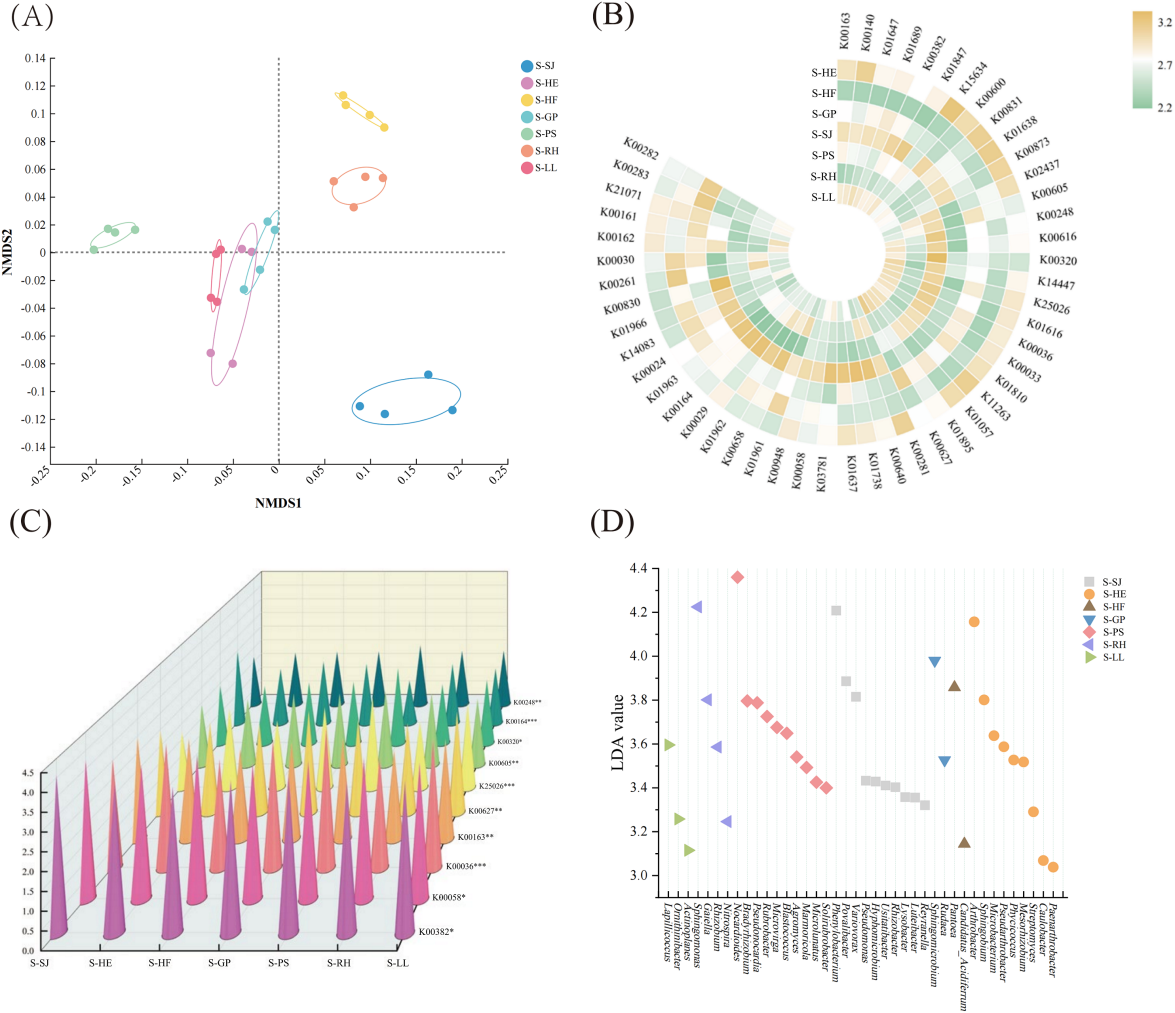
Genes and microorganisms related to carbon decomposition. **(A)** represents NMDS analysis; **(B)** represents the relative abundance of carbon decomposition genes in the rhizosphere soils of different plants; **(C)** represents the difference of carbon decomposition genes; **(D)** represents bacteria related to carbon decomposition (LDA > 3) in the rhizosphere soils of different plants and the unidentified taxa were removed.

To compare the distribution of carbon decomposition genes among rhizosphere soil microorganisms from different plant species, NMDS analysis were performered, which revealed significant differences in carbon decomposition genes among different plant rhizosphere soil microorganisms (ANOSIM, p = 0.001) (**Figure 5A**). The relative abundances of carbon-decomposing genes S-HE, S-SJ, S-LL and S-RH were relatively high, while those of S-PS and S-HF were relatively low. S-GP showed a moderate relative abundance (**Figure 5B**; **Supplementary Table 5**). Among the top 10 carbon decomposition genes with relatively high abundance and significant differences, the relative abundance of S-LL is relatively high, while that of S-HF is relatively low (**Figure 5C**).

A total of 1,059 bacterial genera and 33 fungal genera were found to be involved in carbon decomposition. To identify the specific microbial taxa contributing significantly to differences in carbon decomposition among the rhizosphere soils, linear discriminant analysis effect size (LEfSe) was performed (LDA score ≥ 3). Specifically S-SJ was characterized by *Reyranella*, *Luteibacter*, *Lysobacter*, *Rhizobacter*, *Usitatibacter*, *Hyphomicrobium*, *Pseudomonas*, *Variovorax*, *Povalibacter*, and *Phenylobacterium*. S-GP was dominated by *Rudaea* and *Sphingomicrobium*, while S-HF was distinguished by Candidatus_Acidiferrum and Pantoea. Furthermore, S-LL, S-RH, S-HE, and S-VR harbored 3, 4, 9 and 10 dominant bacterial genera, respectively (**Figure 5D**; **Supplementary Table 6**). In contrast to bacteria, only three fungal genera showed significant differential effects on carbon decomposition. *Aspergillus* was enriched in the rhizosphere of S-SJ, whereas *Darwinula* and *Rhynchospora* were predominantly found in S-HE (**Supplementary Table 7**).

**Figure 6:**
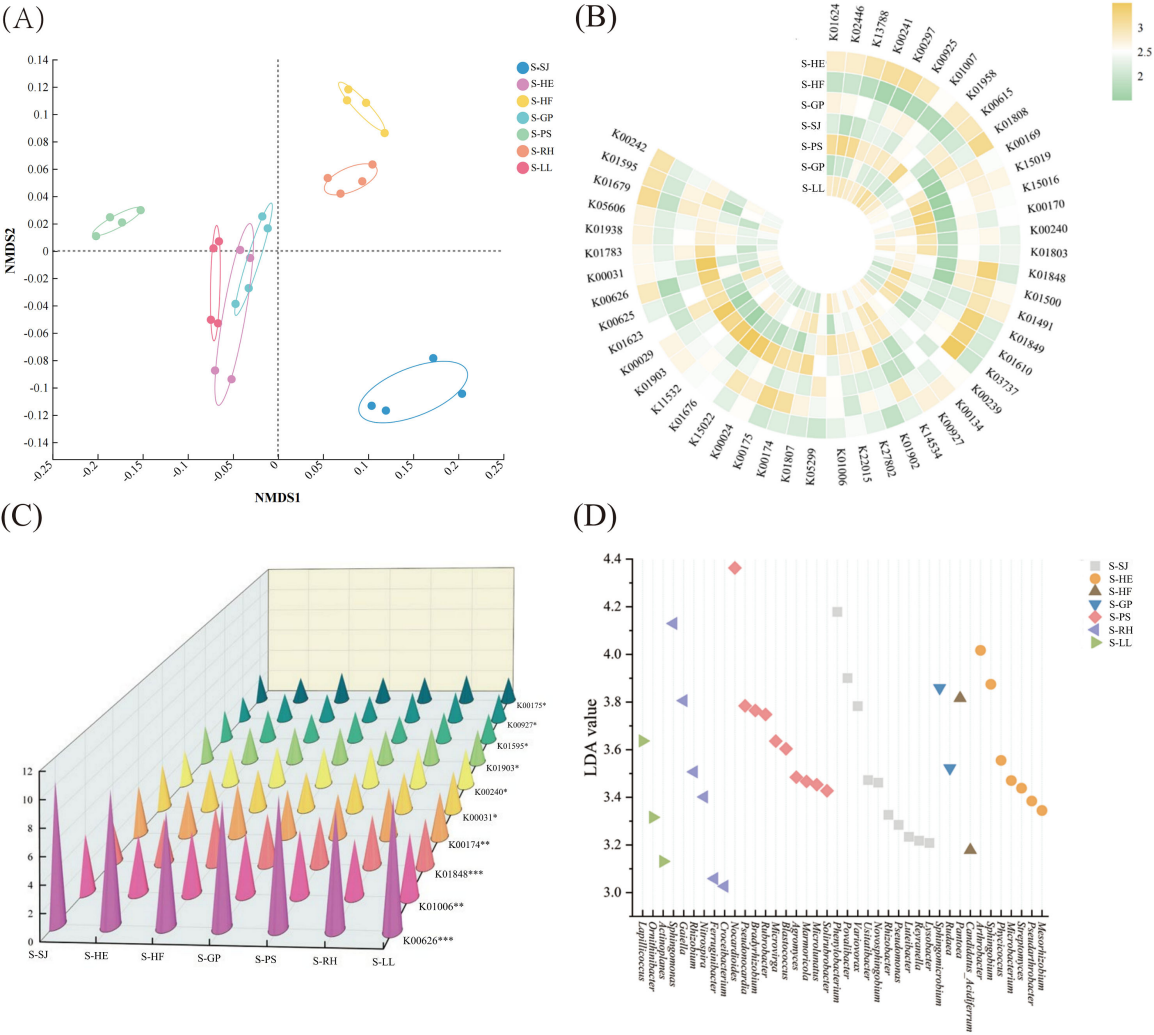
Genes and microorganisms related to carbon fixation. **(A)** represents NMDS analysis; **(B)** represents the relative abundance of carbon fixation genes in the rhizosphere soils of different plants; **(C)** represents the difference of carbon fixation genes; **(D)** represents bacteria related to carbon fixation (LDA > 3) in the rhizosphere soils of different plants, and the unidentified taxa were removed.

NMDS metagenomic analysis revealed significant differences in carbon fixation genes among rhizosphere soil microorganisms from different herbaceous plants (ANOSIM, p = 0.001) (**Figure 6A**). Analysis of the top 50 carbon-immobilization genes in terms of soil microbial abundance in the rhizosphere of plants revealed that the top three relative abuntubers of carbon-immobilization genes were S-HE, S-LL, and S-PS, with S-SJ being the lowest, and S-GP showing moderate abundance (**Figure 6B**; **Supplementary Table 8**). After screening for carbon-fixing genes with significant differences and the top ten relative abundances, it was found that ACAT and atoB (K00626) had relatively high relative abundances in each component, indicating that these genes are dominant for carbon fixation. However, the relative abundance of korB, oorB, oforB (K00175) and pgk (K00927) is relatively low.A total of 815 bacterial genera and 23 fungal genera participated in carbon fixation. LEfSe analysis of the bacteria involved in carbon fixation (LDA ≥ 3) revealed that the dominant bacterial species contained in S-SJ, S-HE, S-HF, S-GP, S-PS, S-RH, and S-LL were 10, 7, 2, 2, 10, 6, and 3, respectively. Among them, the dominant bacteria of S-GP are *Rudaea* and *Sphingomicrobium*; The dominant bacteria of S-HE are *Mesorhizobium*, *Pseudarthrobacter*, *Streptomyces*, *Microbacterium*, *Phycicoccus*, *Sphingobium* and *Arthrobact*er (**Figure 6D**; **Supplementary Table 9**). All 10 bacterial genera present in S-PS exhibited LDA values greater than 3.7. For fungi, only one fungal genus, *Rhynchospora*, was present in S-PS with an LDA value greater than 5.3 (**Table 1**).

### 3.5 Correlation analysis between environmental factors and microorganisms

**Figure 7:**
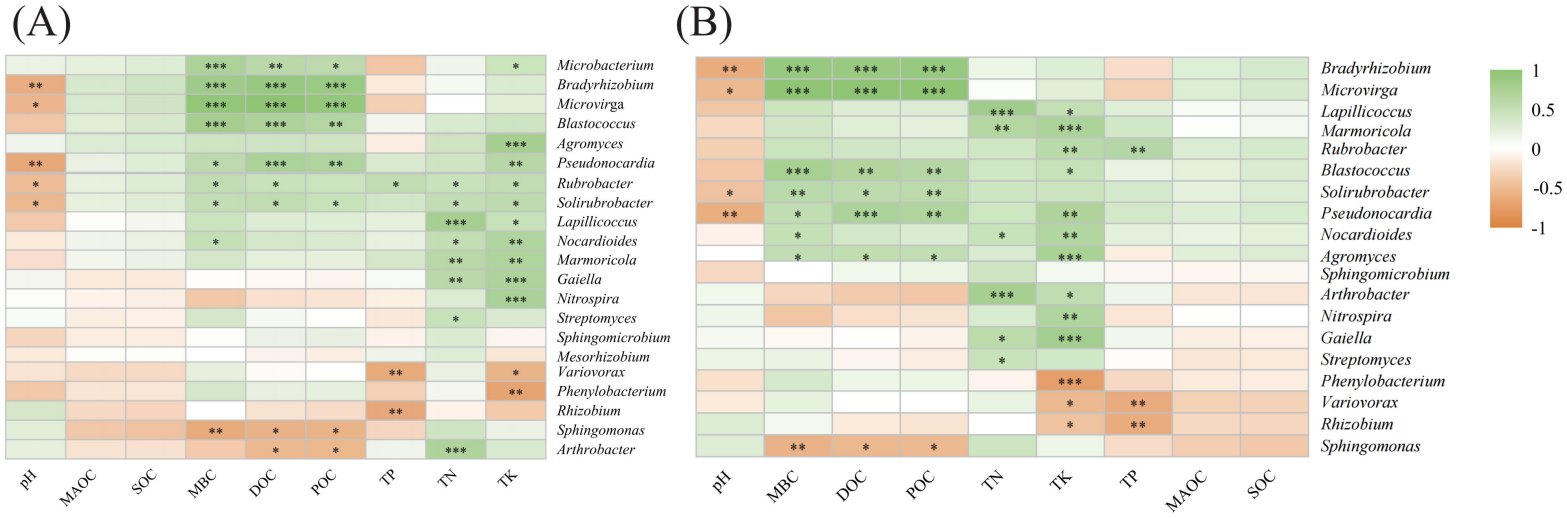
Correlations between environmental factors and microorganisms. **(A)** represents microorganisms related to carbon metabolism, **(B)** represents microorganisms related to carbon fixation. Unidentified taxa were removed. The symbols *, **, and *** indicate statistically significant correlations as follows: * p < 0.05, ** p < 0.01, *** p < 0.001.

To understand the correlations between environmental factors and microorganisms, correlation analysis was performed. The results revealed that, most microorganisms involved in carbon cycling showed no significant correlation with either SOC or MAOC. However, the majority of microorganisms involved in the carbon cycle are significantly positively correlated with MBC, DOC, POC, TN, and TK. Through comparison, it was found that some microorganisms were significantly positively correlated with TN and TK, such as Rubrobacter, Solirubrobacier, Lapillicoccus, Nocardioides, and Marmoricola, indicating that microorganisms involved in the carbon cycle prefer eutrophic conditions. *Bradyrhizobium*, *Microvirga*, *Pseudonocardia*, *Rubrobacter*, and *Solirubrobacter* are negatively correlated with pH and positively correlated with activated carbon components, indicating that these microorganisms may be acid-resistant carbon-fixing bacteria. Most of these microorganisms are involved in both carbon decomposition and carbon fixation simultaneously, but only *Microbacterium* and *Mesorhizobium* are only involved in carbon decomposition. (**Figure 7**; **Table 1**; **Supplementary Table S12**).

## 4 Discussion

### 4.1 Interspecific differences in carbon sequestration capacity

Our results demonstrate that *Gaillardia pulchella* Foug. (P-GP) and *Salvia japonica* Thunb. (P-SJ) have substantially higher carbon assimilation than *Lycopus lucidus* Turcz. ex Benth. (P-LL) (**Figure 2**). This finding is consistent with a previous report on Salvia species, which showed high carbon assimilation levels associated with their robust photosynthetic machinery (Hongshuang et al., 2024). However, P-LL exhibited the lowest fixation capacity among the seven species tested.

A possible explanation for these differences lies in root system architecture. P-GP and P-SJ develop a taproot system that grows deep into the soil, whereas P-LL possesses a rhizomatous root system with shallow, horizontal expansion. Recent studies have shown that deep-rooted plants transport a larger proportion of photosynthetically fixed carbon to deeper soil layers, where lower microbial activity and oxygen availability slow down organic matter decomposition, thereby enhancing long-term carbon storage (A et al., 2024; Sarah et al., 2024). In addition, deep rooting improves access to water and nutrients during dry periods, which can sustain higher photosynthetic rates and, consequently, higher daily CO_2_ fixation.

### 4.2 Interspecific differences in rhizosphere soil carbon and nutrient pools

We also found that there were no significant differences in SOC and MAOC among rhizosphere soils of different plant species, while MBC, DOC, and POC showed significant differences (**Table 1**). According to the results of the Second National Soil Survey of China documented in Soil Species of China (1995), the average SOC content in soils of Jilin Province was 17.66 g/kg (Yue et al., 2022). The SOC contents in rhizosphere soils of all seven plant species in this study were higher than the average SOC content of Jilin Province, suggesting that vegetation establishment increases SOC content through contributions from aboveground litter, root exudates, and root residues (K. et al., 2016). MBC represents the most active and functionally important component of the active carbon pool (Chang and Zhang, 2023). Our results showed a distinct pattern among species. The MBC content in RS-SJ rhizosphere soil (707.87 ± 1.154) was significantly higher than in all other groups, coinciding with the highest DOC and POC values also observed in RS-SJ (**Table 1**). This supports the finding that DOC can stimulate microbial activity and thereby increase MBC (Peiyin et al., 2023). Together, these observations indicate that the rhizosphere of RS-SJ supplies abundant labile carbon sources, creating favorable microhabitats for microbial growth and proliferation. In contrast, RS-HF exhibited the lowest MBC (312.83 ± 0.05), along with the lowest DOC and POC. This consistent pattern suggests that the rhizosphere environment of RS-HF provides limited labile carbon, which may constrain microbial proliferation or impose physiological stress on the microbial community.

Although soil pH varied only within a narrow range (7.33–7.53), significant differences were detected. pH can influence nutrient availability and microbial enzyme activity, potentially exerting selective pressure on microbial community composition. This is consistent with the high inter-group separation observed in our PCoA analysis, suggesting that even small pH variations within the rhizosphere can shape microbial assemblages.

### 4.3 Interspecific differences and trade-offs of carbon-cycling microorganisms in the rhizosphere of different plants

Relevant studies have shown that microorganisms play a crucial role in soil carbon cycling (Audette et al., 2021). This study found that rhizosphere soil microbial alpha and beta were influenced by different plants, with S-SJ exhibiting significantly higher alpha diversity than other groups (**Figure 3**), likely due to the presence of Usitatibacter in its rhizosphere microorganisms. Some species within this family are nitrogen-fixing bacteria capable of converting atmospheric nitrogen into plant-available ammonia, thus improving nitrogen utilization efficiency to enhance soil microbial diversity (Zeyu et al. 2023). This study found that the dominant microorganisms varied among different rhizosphere soils (**Figure 4**). Such in S-SJ *Phenylobacterium* can degrade aromatic compounds and the aromatic compounds produced by other microorganisms degrading lignin can provide carbon sources for them (Singh et al., 2022). In S-GP, *Sphingomicrobium* is typically involved in the degradation of aromatic compounds, may play a role in carbon cycling and bioremediation, and can also adapt to oligotrophic conditions (Shi et al., 2025). In S-PS, *Nocardioides* possesses strong organic matter degradation capabilities, particularly excelling at degrading various recalcitrant pollutants (Ma et al., 2023). In S-RH, *Sphingomonas* plays an important role in carbon and nitrogen metabolism and can promote plant growth through multiple pathways including hormone synthesis, nutrient provision, and microecological regulation (Cordovez et al., 2018; Wang et al., 2022). Although the carbon sequestration capacity of *Pseudolysimachion spicatum* (L.) Opiz (PS) is not strong, the contents of DOC, POC and MBC in its rhizosphere soil are relatively high, which may be related to its unique rhizosphere microbial composition. The dominant microorganisms in its rhizosphere soil include Nocardioides and Rubrobacter. Both of these microorganisms can survive in infertile soil and have a considerable capacity for carbon decomposition (Isabel et al., 2023; Yecheng et al., 2023) (**Figure 6**). The enrichment of these microorganisms is not random, but rather reflects a match between their physiological traits and the chemical environment of the corresponding rhizosphere (carbon source types, etc.), representing an adaptation to the environment.

### 4.4 Coupling of rhizosphere microorganisms with active carbon pools and nutrients

Microorganisms play a core driving role in soil organic carbon transformation (Xingping et al.; Malik et al., 2018; Naylor et al., 2020). This study found that rhizosphere microbial communities showed no significant correlation with SOC and MAOC, but were significantly positively correlated with DOC, POC, MBC, TN, TP, and TK (**Figure 7**). This result suggests that the rhizosphere microbial community primarily tends to utilize readily available carbon sources rather than recalcitrant carbon, while actively participating in soil nutrient cycling through processes such as nitrogen fixation, phosphate solubilization, and mineral weathering. Genera such as Bradyrhizobium and Microvirga, which were highly positively correlated with labile carbon pools (DOC, POC), are likely typical copiotrophic bacteria (Stone et al., 2021). They grow rapidly, require large amounts of available carbon sources, and tend to maintain high activity under slightly acidic conditions (negatively correlated with pH). In contrast, Sphingomonas showed a negative correlation with these labile carbon components. Sphingomonas is known to possess a strong metabolic capacity for degrading recalcitrant organic compounds, including complex aromatic compounds and polycyclic aromatic hydrocarbons (Lin et al., 2012). Its negative correlation implies a competitive exclusion mechanism: when DOC and POC are abundant, copiotrophic bacteria proliferate and dominate; conversely, when available carbon becomes scarce, oligotrophic or specialized metabolic bacteria such as Sphingomonas gain a competitive advantage by decomposing recalcitrant carbon sources (e.g., the recalcitrant fraction of SOC). Microbial carbon-cycling genes play a critical role in carbon transformation (Zhou et al., 2025). Studies have shown that certain microorganisms (e.g., Pantoea) are involved in both carbon fixation and carbon decomposition, indicating that these microbes possess both carbon-fixation and carbon-decomposition genes. The processes of carbon fixation and carbon decomposition are closely linked. This finding is consistent with the results reported by Pingping et al.: both Pseudomonas and Achromobacter possess the capacity for hydrocarbon degradation and carbon fixation (Cai et al., 2025).

The Spearman correlation analysis demonstrated that the microbial oxidative phosphorylation pathway was significantly and negatively correlated with plant photosynthetic and carbon sequestration indicators, including Pn, WCO_2_, and wCO_2_ (**Supplementary Figure S1**). This consistent negative linkage firmly establishes a multi-scale coupling between aboveground carbon fixation and belowground microbial energetic strategies. Higher Pn, along with elevated WCO_2_ and wCO_2_, denotes a robust plant physiological status and a substantial influx of labile, belowground carbon inputs (such as simple carbohydrates and organic acids). Under such carbon-replete conditions, the rhizomicrobial community tends to prioritize efficient anabolic pathways and biomass accumulation, thereby dampening the necessity for highly energetic catabolic pathways like oxidative phosphorylation. Conversely, when plant photosynthesis and carbon sequestration were suppressed, the reduction in readily available carbon sources likely forced the microbial community to rely on heightened respiratory and metabolic efficiency upregulating oxidative phosphorylation—to extract energy from more recalcitrant soil organic matter for cellular maintenance. This co-response pattern further supports the ecological partition of r/K selection, suggesting that the magnitude of plant-derived carbon inputs dynamically governs the functional and energetic prioritization of the soil microbiome.

## 5 Conclusion

Through a metagenomic study on the carbon sequestration capacity of seven plant species and their rhizosphere soil microorganisms, the following key findings were obtained: different plant species exhibit distinct carbon sequestration capacities, among which *Gaillardia pulchella* Foug. and *Salvia jponica* Thunb. show significantly higher wCO_2_ and WCO_2_ values than the other tested plants. Specifically, this study systematically explored the carbon sequestration potential of seven plant species and their associated rhizosphere soil microorganisms using metagenomic approaches. The results demonstrated significant differences in carbon sequestration capacity across different plant species, with *Gaillardia pulchella* Foug. and *Salvia jponica* Thunb. displaying superior carbon sequestration potential compared to the other plants. More importantly, our study revealed that some rhizosphere soil microorganisms are simultaneously involved in both carbon decomposition and carbon fixation processes. This discovery confirms that microbial catabolism and anabolism are not isolated from each other but are closely coupled through a complex metabolic network. Such interaction is presumably the intrinsic mechanism that drives soil nutrient cycling, enhances soil fertility, and provides effective nutritional support for plant growth. The sampling in this study was conducted in August, which coincides with the peak growing season of plants in Northeast China. However, a single time-point approach cannot reveal the dynamic succession of rhizosphere microbial communities and their carbon-cycling functions across plant phenological stages. Therefore, future studies should employ multi time point continuous sampling to monitor the temporal changes in plant carbon fixation capacity, soil carbon pool components (e.g., SOC, MBC, POC), and the abundances of microbial carbon decomposition and carbon fixation genes, thereby providing a more comprehensive understanding of the phenological response mechanisms of rhizosphere carbon cycling.

## Data Availability

The datasets presented in this study can be found in online repositories. The names of the repository/repositories and accession number(s) can be found in the article/**Supplementary Material**.
