# Supplementary material for: Differences in carbon sequestration capacity, rhizosphere microorganisms and metabolic functions among different herbaceous plants
Source: Front Plant Sci. 2026 Jun 17;17:1849153. doi: 10.3389/fpls.2026.1849153 (PMC13320889; doi:10.3389/fpls.2026.1849153)
Supplement: Supplementary file 1 [file Table1.docx]

**Differences in Carbon Sequestration Capacity, Rhizosphere Microorganisms and Metabolic Functions Among Different Herbaceous Plants**

**Yu Zhou^1^, Pushun Bian^1^, Chen Yang^2^, Jiayan Qu^2^, Huaying Wang^2^,Wei Gao^1,*^**

^1^College of Landscape Architecture, Changchun University, Changchun, Jilin Province, 130022, China.

^2^School of Life Sciences, Northeast Normal University, Changchun, Jilin Province, 130024, China.

*Correspondence:

Wei Gao

gaowei080105@126.com

Attached table

**Table S1** Bacterial Alpha Diversity Values

| **estimators** | 1. **LL-mean** | **S-LL-sd** | **S-RH-mean** | **S-RH-sd** | **S-PS-mean** | **S-PS-sd** | **S-SJ-mean** | **S-SJ-sd** | **S-GP-mean** | **S-GP-sd** | **S-HF-mean** | **S-HF-sd** | **S-HE-mean** | **S-HE-sd** |
| --- | --- | --- | --- | --- | --- | --- | --- | --- | --- | --- | --- | --- | --- | --- |
| ace | 2174 | 26.22975 | 2235 | 15.64182 | 2167.5 | 22.21861 | 2258.75 | 41.84396 | 2185.75 | 39.63479 | 2222 | 45.30636 | 2171.25 | 57.61583 |
| shannon | 4.4494 | 0.01129 | 4.50383 | 0.0163 | 4.36345 | 0.01769 | 4.7234 | 0.01747 | 4.49598 | 0.03908 | 4.45589 | 0.02871 | 4.49864 | 0.01779 |

**Table S2** Fungal Alpha Diversity Values

| **estimators** | **S-LL-mean** | **S-LL-sd** | **S-RH-mean** | **S-RH-sd** | **S-PS-mean** | **S-PS-sd** | **S-SJ-mean** | **S-SJ-sd** | **S-GP-mean** | **S-GP-sd** | **S-HF-mean** | **S-HF-sd** | **S-HE-mean** | **S-HE-sd** |
| --- | --- | --- | --- | --- | --- | --- | --- | --- | --- | --- | --- | --- | --- | --- |
| ace | 28.5 | 4.65475 | 93.5 | 9.57427 | 53.25 | 4.92443 | 35.25 | 0.95743 | 31.5 | 3.10913 | 34.75 | 3.59398 | 26.25 | 1.25831 |
| shannon | 2.31183 | 0.2044 | 3.19601 | 0.05191 | 3.10383 | 0.15793 | 2.58102 | 0.10249 | 2.36377 | 0.10862 | 2.53691 | 0.07204 | 2.21769 | 0.05217 |

**Table S3** LDA Values of Bacteria

| **Group** | **Species name** | **LDA_value** | **Group** | **Species name** | **LDA_value** | **Group** | **Species name** | **LDA_value** |
| --- | --- | --- | --- | --- | --- | --- | --- | --- |
| S-SJ | unclassified_Verrucomicrobiaceae | 3.6000 | S-RH | Nitrospira | 3.3559 | S-LL | Variovorax | 3.8662 |
|  | Luteibacter | 3.5017 |  | unclassified_Chitinophagaceae | 4.0891 |  | Actinoplanes | 3.1309 |
|  | Usitatibacter | 3.2485 |  | unclassified_Candidatus_Binatia | 3.3527 |  | Ornithinibacter | 3.2490 |
|  | Rhizobacter | 3.4028 | S-PS | unclassified_Thermoleophilaceae | 3.7714 | S-HE | Phycicoccus | 3.5813 |
|  | unclassified_Acidimicrobiia | 3.0222 |  | Mycobacterium | 3.2832 |  | Paenarthrobacter | 3.0966 |
|  | Lysobacter | 3.1918 |  | Blastococcus | 3.5490 |  | Arthrobacter | 4.1576 |
|  | unclassified_Betaproteobacteria | 3.0446 |  | unclassified_Rubrobacteraceae | 3.7227 |  | Mesorhizobium | 3.4929 |
|  | Novosphingobium | 3.2139 |  | Agromyces | 3.5611 |  | Gaiella | 3.7323 |
|  | unclassified_Pseudomonadota | 3.5533 |  | Microvirga | 3.8301 |  | Microbacterium | 3.4779 |
|  | Aquincola | 3.1913 |  | unclassified_Xanthobacteraceae | 3.3796 |  | Sphingobium | 3.8835 |
|  | unclassified_Burkholderiaceae | 3.8703 |  | Pseudolysinimonas | 3.2978 |  | Pseudarthrobacter | 3.6716 |
|  | Reyranella | 3.1317 |  | unclassified_Actinomycetota | 4.0069 |  | unclassified_Gemmatimonadales | 3.7608 |
|  | unclassified_Steroidobacteraceae | 4.0239 |  | Microlunatus | 3.4559 |  | unclassified_Casimicrobiaceae | 3.2850 |
|  | unclassified_Micropepsaceae | 3.1462 |  | Nocardioides | 4.3228 | S-HF | unclassified_Candidatus_Rokuibacteriota | 3.3105 |
|  | unclassified_Caulobacteraceae | 3.1685 |  | unclassified_Candidatus_Limnocylindrales | 3.6537 |  | unclassified_Dongiaceae | 3.3985 |
|  | Pseudomonas | 3.6286 |  | unclassified_Kineosporiaceae | 3.0913 |  | Pantoea | 3.9843 |
|  | Hyphomicrobium | 3.2164 |  | unclassified_Propionibacteriaceae | 4.1958 |  | unclassified_Candidatus_Limnocylindria | 3.3278 |
|  | unclassified_Gammaproteobacteria | 3.0460 |  | unclassified_Nocardioidaceae | 3.8356 |  | unclassified_Chloroflexota | 3.6128 |
|  | Phenylobacterium | 4.2113 |  | Pseudonocardia | 3.7186 |  | unclassified_Gemmatimonadaceae | 3.7508 |
|  | Povalibacter | 4.0218 |  | Streptomyces | 3.3761 |  | unclassified_Acidobacteriota | 4.1841 |
|  | unclassified_Beijerinckiaceae | 3.2438 |  | Rubrobacter | 3.9459 |  | unclassified_Burkholderiales | 3.6622 |
|  | unclassified_Ilumatobacteraceae | 3.1814 |  | unclassified_Myxococcia | 3.1532 |  | Candidatus_Acidiferrum | 3.0561 |
|  | Piscinibacter | 3.0154 |  | Nakamurella | 3.0947 |  | unclassified_Candidatus_Methylomirabilota | 3.7562 |
|  | unclassified_Alphaproteobacteria | 3.2897 |  | unclassified_Solirubrobacterales | 3.7882 |  | unclassified_Gemmatimonadota | 3.0139 |
|  | unclassified_Hyphomicrobiaceae | 3.1262 |  | Marmoricola | 3.3421 |  | unclassified_Bacteroidota | 3.1304 |
| S-RH | unclassified_Sphingomonadales | 3.1111 |  | Bradyrhizobium | 4.1597 |  | unclassified_Kofleriaceae | 3.3192 |
|  | Sphingomonas | 4.2232 | S-LL | Solirubrobacter | 3.3333 |  | unclassified_Pyrinomonadaceae | 4.2656 |
|  | unclassified_Vicinamibacterales | 4.3536 |  | unclassified_Solirubrobacteraceae | 4.1060 |  | unclassified_d__Bacteria | 3.0241 |
|  | Rhizobium | 3.7287 |  | Lapillicoccus | 3.5877 | S-GP | Sphingomicrobium | 3.9007 |
|  | Ferruginibacter | 3.1396 |  | unclassified_Gaiellales | 3.1382 |  | Rudaea | 3.6596 |
|  | Chryseolinea | 3.0268 |  | unclassified_Gaiellaceae | 3.9045 |  | unclassified_Actinomycetes | 3.7161 |
|  |  |  |  |  |  |  | unclassified_Mycobacteriales | 3.2172 |

**Table S4** Attached table D lefse Values of Fungi

| **Group** | **Species name** | **LDA_value** | **Group** | **Species name** | **LDA_value** | **Group** | **Species name** | **LDA_value** |
| --- | --- | --- | --- | --- | --- | --- | --- | --- |
| S-SJ | Lichtheimia | 4.3483 | S-PS | Pichia | 4.1796 | S-HE | Quaeritorhiza | 4.5133 |
|  | Ambispora | 4.4081 |  | Mortierella | 4.2856 | S-HF | Tuber | 4.1994 |
|  | Aureobasidium | 4.9109 |  | Fusarium | 4.2532 |  | Rhizopus | 5.1627 |
| S-RH | Paraglomus | 4.4710 | S-LL | Colletotrichum | 4.3722 |  | Ceratobasidium | 4.2250 |
| S-PS | Metschnikowia | 4.3613 |  | Aspergillus | 4.9972 |  | Penicillium | 4.4233 |
|  |  |  | S-HE | Friedmanniomyces | 4.7075 | S-GP | Verticillium | 4.2544 |

**Table S5** Carbon Gene Abundance

|  | **S-SJ** | **S-RH** | **S-HF** | **S-PS** | **S-GP** | **S-LL** | **S-HE** |  | **S-SJ** | **S-RH** | **S-HF** | **S-PS** | **S-GP** | **S-LL** | **S-HE** |
| --- | --- | --- | --- | --- | --- | --- | --- | --- | --- | --- | --- | --- | --- | --- | --- |
| K01895 | 3.2189 | 3.2154 | 3.2251 | 3.2263 | 3.2318 | 3.2440 | 3.2307 | K00261 | 2.5453 | 2.7030 | 2.7235 | 2.6078 | 2.6448 | 2.6063 | 2.6093 |
| K00036 | 3.0587 | 3.0457 | 3.0292 | 3.1554 | 3.1211 | 3.1317 | 3.1202 | K01847 | 2.7228 | 2.6111 | 2.5347 | 2.6472 | 2.6177 | 2.6630 | 2.6602 |
| K00382 | 3.1867 | 3.1385 | 3.1241 | 3.1650 | 3.1638 | 3.1677 | 3.1614 | K25026 | 2.8128 | 2.8667 | 2.8695 | 3.0106 | 2.9660 | 2.9928 | 2.9735 |
| K00058 | 3.1486 | 3.1302 | 3.1385 | 3.1292 | 3.1288 | 3.1383 | 3.1342 | K11263 | 2.5365 | 2.5849 | 2.5411 | 2.7506 | 2.6774 | 2.7470 | 2.7972 |
| K00627 | 2.9648 | 2.9811 | 2.9760 | 3.0055 | 3.0502 | 3.0543 | 3.0100 | K01616 | 2.6838 | 2.6921 | 2.6319 | 2.8795 | 2.7789 | 2.8384 | 2.8171 |
| K01647 | 3.0419 | 2.9975 | 2.9770 | 3.0167 | 3.0265 | 3.0425 | 3.0267 | K00164 | 2.9682 | 2.8600 | 2.8756 | 2.7227 | 2.8763 | 2.8475 | 2.8486 |
| K00163 | 3.0467 | 2.9631 | 2.9653 | 3.0277 | 3.0189 | 3.0342 | 3.0440 | K00033 | 2.6585 | 2.6594 | 2.6401 | 2.7473 | 2.7140 | 2.7272 | 2.7225 |
| K00029 | 2.7172 | 2.5947 | 2.5316 | 2.3719 | 2.5422 | 2.5478 | 2.6005 | K00605 | 2.8625 | 2.9149 | 2.9077 | 2.9351 | 2.9286 | 2.9008 | 2.9025 |
| K15634 | 2.3858 | 2.3796 | 2.3512 | 2.4198 | 2.3855 | 2.4314 | 2.4756 | K00320 | 2.8569 | 2.8448 | 2.8552 | 2.9585 | 2.8776 | 2.8974 | 2.8546 |
| K14447 | 2.4519 | 2.3666 | 2.4014 | 2.5558 | 2.4806 | 2.5473 | 2.4963 | K01961 | 2.7840 | 2.8003 | 2.8078 | 2.6732 | 2.7604 | 2.7208 | 2.7319 |
| K01057 | 2.3685 | 2.4161 | 2.3809 | 2.4782 | 2.4739 | 2.4867 | 2.4950 | K00281 | 2.7507 | 2.7406 | 2.6891 | 2.6788 | 2.6918 | 2.6998 | 2.7645 |
| K00658 | 2.6169 | 2.5179 | 2.4943 | 2.2184 | 2.4493 | 2.4525 | 2.4423 | K21071 | 2.7155 | 2.7119 | 2.7328 | 2.6882 | 2.7410 | 2.7130 | 2.7299 |
| K02437 | 2.4757 | 2.5570 | 2.5360 | 2.5732 | 2.5166 | 2.5175 | 2.5703 | K00024 | 2.7488 | 2.7101 | 2.7262 | 2.6699 | 2.7124 | 2.6776 | 2.7118 |
| K00283 | 2.5006 | 2.5352 | 2.5495 | 2.5216 | 2.5879 | 2.5481 | 2.5326 | K01738 | 2.8424 | 2.7275 | 2.7324 | 2.6807 | 2.7475 | 2.7607 | 2.7808 |
| K00030 | 2.4057 | 2.5238 | 2.5577 | 2.4849 | 2.5357 | 2.4923 | 2.4967 | K03781 | 2.8217 | 2.7132 | 2.7448 | 2.6996 | 2.6888 | 2.7622 | 2.7517 |
| K00282 | 2.4789 | 2.4926 | 2.4955 | 2.4710 | 2.5468 | 2.5070 | 2.4975 | K01966 | 2.8430 | 2.8007 | 2.8350 | 2.7919 | 2.8111 | 2.8219 | 2.7987 |
| K00162 | 2.5424 | 2.4779 | 2.5123 | 2.4822 | 2.5318 | 2.5328 | 2.5348 | K00948 | 2.8152 | 2.7828 | 2.7928 | 2.7865 | 2.7869 | 2.7849 | 2.8050 |
| K00161 | 2.5190 | 2.4887 | 2.5117 | 2.4729 | 2.5419 | 2.5407 | 2.5288 | K01810 | 2.7433 | 2.7374 | 2.7499 | 2.7946 | 2.7906 | 2.7820 | 2.7931 |
| K00831 | 2.5778 | 2.5152 | 2.5378 | 2.5256 | 2.5571 | 2.5692 | 2.6115 | K01638 | 2.8040 | 2.7449 | 2.7431 | 2.7548 | 2.7765 | 2.7834 | 2.8249 |
| K14083 | 2.5834 | 2.5192 | 2.5687 | 2.5140 | 2.5491 | 2.4974 | 2.5171 | K00873 | 2.7878 | 2.7501 | 2.7476 | 2.7633 | 2.7620 | 2.7682 | 2.7940 |
| K01963 | 2.5408 | 2.4396 | 2.4459 | 2.2510 | 2.4395 | 2.3880 | 2.4216 | K00140 | 2.8481 | 2.7649 | 2.7528 | 2.8189 | 2.8117 | 2.8455 | 2.8787 |
| K01962 | 2.5010 | 2.4626 | 2.4407 | 2.2314 | 2.4065 | 2.3742 | 2.3791 | K00600 | 2.8697 | 2.8377 | 2.8223 | 2.8440 | 2.8239 | 2.8450 | 2.8674 |
| K01637 | 2.5574 | 2.4453 | 2.4539 | 2.4162 | 2.4990 | 2.4641 | 2.5171 | K01689 | 2.8475 | 2.8181 | 2.7942 | 2.8208 | 2.8417 | 2.8429 | 2.8375 |
| K00640 | 2.5526 | 2.5068 | 2.4382 | 2.4073 | 2.4377 | 2.4222 | 2.4795 | K00248 | 2.8003 | 2.8280 | 2.8190 | 2.8793 | 2.8404 | 2.8513 | 2.8395 |
| K00830 | 2.5775 | 2.4498 | 2.4691 | 2.4128 | 2.4221 | 2.4203 | 2.4008 | K00616 | 2.7611 | 2.7982 | 2.8122 | 2.8575 | 2.8203 | 2.8398 | 2.8233 |

**Table S6** shows the LDA values of carbon-degrading bacteria

| **Group** | **Species name** | **LDA_value** | **Group** | **Species name** | **LDA_value** | **Group** | **Species name** | **LDA_value** |
| --- | --- | --- | --- | --- | --- | --- | --- | --- |
| S-GP | unclassified_Actinomycetes | 3.63976 | S-LL | unclassified_Gaiellales | 3.26783 | S-SJ | unclassified_Burkholderiaceae | 3.83587 |
|  | Sphingomicrobium | 3.98023 |  | unclassified_Gaiellaceae | 3.96826 |  | unclassified_Ilumatobacteraceae | 3.54919 |
|  | unclassified_Mycobacteriales | 3.33922 |  | Actinoplanes | 3.11510 |  | Lysobacter | 3.35820 |
|  | Rudaea | 3.52666 |  | Ornithinibacter | 3.25795 |  | Rubrivivax | 3.09278 |
| S-HF | unclassified_Pyrinomonadaceae | 4.04452 | S-RH | unclassified_Sphingomonadales | 3.15756 |  | unclassified_Hyphomicrobiaceae | 3.24771 |
|  | Pantoea | 3.85817 |  | Sphingomonas | 4.22483 |  | unclassified_Betaproteobacteria | 3.15145 |
|  | unclassified_Candidatus_Dormiibacterota | 3.14924 |  | unclassified_Chloroflexota | 3.80848 |  | unclassified_Acidimicrobiia | 3.13315 |
|  | unclassified_Burkholderiales | 3.81522 |  | Rhizobium | 3.58610 |  | Piscinibacter | 3.26492 |
|  | unclassified_Dongiaceae | 3.50759 |  | Gaiella | 3.80129 |  | Pseudomonas | 3.43341 |
|  | unclassified_Gemmatimonadaceae | 3.73881 |  | unclassified_Vicinamibacterales | 4.28758 | S-PS | Pseudonocardia | 3.78759 |
|  | unclassified_Verrucomicrobiia | 3.13973 |  | Nitrospira | 3.24612 |  | Microvirga | 3.67553 |
|  | unclassified_Candidatus_Limnocylindria | 3.37839 |  | unclassified_Chitinophagaceae | 3.85534 |  | unclassified_Myxococcia | 3.36937 |
|  | unclassified_Acidobacteriota | 4.02375 | S-SJ | unclassified_Alphaproteobacteria | 3.33928 |  | Agromyces | 3.54077 |
|  | unclassified_Candidatus_Rokuibacteriota | 3.43735 |  | unclassified_Aestuariivirgaceae | 3.05231 |  | Nakamurella | 3.34432 |
|  | unclassified_Anaerolineales | 3.63564 |  | unclassified_Micropepsaceae | 3.05045 |  | Marmoricola | 3.49314 |
|  | unclassified_Kofleriaceae | 3.37191 |  | Luteibacter | 3.35582 |  | unclassified_Solirubrobacterales | 3.86098 |
|  | Candidatus_Acidiferrum | 3.14469 |  | Aquincola | 3.04142 |  | unclassified_Propionibacteriaceae | 4.04472 |
|  | unclassified_Gemmatimonadota | 3.03939 |  | Nordella | 3.13457 |  | Rubrobacter | 3.72539 |
|  | unclassified_Candidatus_Binatia | 3.35179 |  | unclassified_Steroidobacteraceae | 3.97911 |  | unclassified_Actinomycetota | 4.08730 |
|  | unclassified_Candidatus_Methylomirabilota | 3.91563 |  | Povalibacter | 3.88624 |  | Pseudolysinimonas | 3.29830 |
| S-HE | unclassified_Casimicrobiaceae | 3.43715 |  | unclassified_Verrucomicrobiaceae | 3.54300 |  | Solirubrobacter | 3.40012 |
|  | Caulobacter | 3.06866 |  | unclassified_Beijerinckiaceae | 3.23781 |  | Mycobacterium | 3.25242 |
|  | Arthrobacter | 4.15673 |  | Rhizobacter | 3.40359 |  | Microlunatus | 3.42605 |
|  | Phycicoccus | 3.52699 |  | Reyranella | 3.32108 |  | unclassified_Nocardioidaceae | 3.88875 |
|  | Paenarthrobacter | 3.03826 |  | Phenylobacterium | 4.20872 |  | Blastococcus | 3.64875 |
|  | Mesorhizobium | 3.51799 |  | Variovorax | 3.81523 |  | unclassified_Solirubrobacteraceae | 4.13765 |
|  | Streptomyces | 3.29049 |  | unclassified_Pseudomonadota | 3.54660 |  | unclassified_Thermoleophilaceae | 3.87584 |
|  | Sphingobium | 3.80095 |  | Usitatibacter | 3.41138 |  | unclassified_Rubrobacteraceae | 3.68291 |
|  | Pseudarthrobacter | 3.58739 |  | Hyphomicrobium | 3.42924 |  | Nocardioides | 4.36014 |
|  | Microbacterium | 3.63782 |  | unclassified_Xanthobacteraceae | 3.34286 |  | unclassified_Candidatus_Limnocylindrales | 3.71294 |
| S-LL | unclassified_Gemmatimonadales | 3.62317 |  | Novosphingobium | 3.30986 |  | Bradyrhizobium | 3.79639 |
|  | Lapillicoccus | 3.59580 |  | unclassified_Gammaproteobacteria | 3.00301 |  |  |  |

**Table S7** shows the LDA values of carbon-degrading fungi

| **Group** | **Species name** | **LDA_value** | **Group** | **Species name** | **LDA_value** |
| --- | --- | --- | --- | --- | --- |
| S-SJ | Aspergillus | 5.1197 | S-HE | Rhynchospora | 5.2896 |
|  |  |  |  | Darwinula | 5.3262 |

**Table S8** Abundance of carbon-fixed genes

|  | **S-SJ** | **S-PS** | **S-GP** | **S-LL** | **S-HE** | **S-RH** | **S-HF** |  | **S-SJ** | **S-PS** | **S-GP** | **S-LL** | **S-HE** | **S-RH** | **S-HF** |
| --- | --- | --- | --- | --- | --- | --- | --- | --- | --- | --- | --- | --- | --- | --- | --- |
| K00626 | 3.3874 | 3.3366 | 3.3164 | 3.3414 | 3.3590 | 3.2906 | 3.2712 | K01676 | 2.5581 | 2.3805 | 2.3990 | 2.4428 | 2.4591 | 2.4818 | 2.4465 |
| K03737 | 2.4367 | 2.3494 | 2.4801 | 2.4034 | 2.3646 | 2.5478 | 2.6251 | K01491 | 2.6065 | 2.6123 | 2.6051 | 2.5915 | 2.6449 | 2.6727 | 2.6584 |
| K00615 | 3.1554 | 3.1751 | 3.1831 | 3.1848 | 3.1888 | 3.1602 | 3.1545 | K01610 | 2.5921 | 2.5529 | 2.6130 | 2.5693 | 2.5719 | 2.6530 | 2.6653 |
| K00239 | 3.0982 | 3.0966 | 3.0909 | 3.1052 | 3.0996 | 3.0987 | 3.0982 | K01783 | 2.5458 | 2.5139 | 2.5259 | 2.5082 | 2.5228 | 2.4876 | 2.5051 |
| K01006 | 3.0072 | 3.0432 | 3.0194 | 3.0382 | 3.0059 | 2.9824 | 3.0199 | K05606 | 2.5049 | 2.4998 | 2.4874 | 2.4894 | 2.5039 | 2.4819 | 2.4772 |
| K27802 | 3.0080 | 3.0114 | 2.9959 | 3.0022 | 2.9976 | 2.9995 | 3.0066 | K01808 | 2.4147 | 2.5179 | 2.5588 | 2.5298 | 2.5865 | 2.4900 | 2.4937 |
| K00174 | 2.9515 | 2.8835 | 2.9194 | 2.9138 | 2.9094 | 2.9454 | 2.9575 | K01624 | 2.5083 | 2.5984 | 2.5621 | 2.5717 | 2.5711 | 2.4742 | 2.4825 |
| K01848 | 2.8539 | 2.9240 | 2.9443 | 2.9242 | 2.8965 | 2.9424 | 2.9876 | K01938 | 2.5749 | 2.5692 | 2.5614 | 2.5434 | 2.5718 | 2.5497 | 2.5427 |
| K01903 | 2.8553 | 2.7949 | 2.8256 | 2.8184 | 2.8327 | 2.8259 | 2.8223 | K00241 | 2.5645 | 2.5973 | 2.5582 | 2.5971 | 2.6130 | 2.5844 | 2.5195 |
| K00031 | 2.9201 | 2.8382 | 2.8403 | 2.8696 | 2.8729 | 2.8420 | 2.8594 | K01803 | 2.5295 | 2.6008 | 2.5734 | 2.5895 | 2.5621 | 2.5778 | 2.5588 |
| K00240 | 2.8196 | 2.8676 | 2.8521 | 2.8570 | 2.8412 | 2.8509 | 2.8554 | K22015 | 1.8259 | 1.8384 | 1.7649 | 1.8256 | 1.7924 | 1.7911 | 1.7477 |
| K00297 | 2.6608 | 2.7023 | 2.6901 | 2.7010 | 2.7286 | 2.6479 | 2.5920 | K01500 | 1.6813 | 1.8331 | 1.8856 | 1.8183 | 1.8613 | 1.8754 | 1.9094 |
| K00927 | 2.6851 | 2.7577 | 2.6943 | 2.7419 | 2.7454 | 2.7613 | 2.7243 | K14534 | 1.7604 | 1.9164 | 1.6711 | 1.8257 | 1.7877 | 1.9219 | 1.9203 |
| K01007 | 2.7347 | 2.7443 | 2.7334 | 2.7649 | 2.7333 | 2.7120 | 2.6850 | K13788 | 2.0723 | 2.3127 | 2.1993 | 2.2705 | 2.2867 | 2.0969 | 2.0312 |
| K01679 | 2.7352 | 2.7064 | 2.7442 | 2.7355 | 2.7644 | 2.6870 | 2.6844 | K15019 | 1.9954 | 2.2924 | 2.1892 | 2.1954 | 2.2176 | 2.1633 | 2.2282 |
| K00024 | 2.7488 | 2.6699 | 2.7124 | 2.6776 | 2.7118 | 2.7101 | 2.7262 | K15022 | 2.3178 | 2.1171 | 2.1859 | 2.1561 | 2.1659 | 2.1841 | 2.2624 |
| K00175 | 2.7653 | 2.7069 | 2.7036 | 2.7118 | 2.6984 | 2.7416 | 2.7785 | K00242 | 2.2096 | 2.1538 | 2.1824 | 2.1835 | 2.2416 | 2.2036 | 2.1639 |
| K01902 | 2.7554 | 2.7299 | 2.7111 | 2.7379 | 2.6940 | 2.7226 | 2.7206 | K01849 | 1.9836 | 2.0720 | 2.0524 | 2.0386 | 2.0123 | 2.1697 | 2.2318 |
| K00134 | 2.7710 | 2.7750 | 2.7771 | 2.7827 | 2.7862 | 2.7818 | 2.7687 | K01807 | 2.1837 | 2.0780 | 2.0572 | 2.0256 | 2.0467 | 2.1596 | 2.1278 |
| K01595 | 2.7846 | 2.6946 | 2.7660 | 2.7440 | 2.7897 | 2.7547 | 2.7189 | K11532 | 2.2226 | 1.9037 | 2.0790 | 2.0702 | 2.0872 | 2.1369 | 2.1099 |
| K01958 | 2.4602 | 2.5616 | 2.4235 | 2.5249 | 2.5156 | 2.3520 | 2.3156 | K00170 | 1.9307 | 2.1365 | 2.0645 | 2.0238 | 2.0458 | 2.0792 | 2.0157 |
| K02446 | 2.3136 | 2.5335 | 2.4492 | 2.4862 | 2.4768 | 2.3575 | 2.3414 | K00625 | 2.0048 | 1.8999 | 1.9565 | 1.9729 | 1.9340 | 2.0009 | 1.9048 |
| K00925 | 2.3304 | 2.4214 | 2.4029 | 2.4298 | 2.4253 | 2.3270 | 2.2706 | K15016 | 1.6931 | 2.1306 | 1.9949 | 2.0092 | 1.9751 | 1.9528 | 2.0208 |
| K01623 | 2.3968 | 2.2778 | 2.3838 | 2.3792 | 2.3476 | 2.3312 | 2.2961 | K05299 | 2.0088 | 2.0289 | 2.0381 | 2.0133 | 1.9672 | 2.0058 | 2.0010 |
| K00029 | 2.7172 | 2.3719 | 2.5422 | 2.5478 | 2.6005 | 2.5947 | 2.5316 | K00169 | 1.8390 | 2.0332 | 2.0166 | 2.0033 | 1.9843 | 1.9920 | 2.0292 |

**Table S9** LDA values of carbon-fixed bacteria

| **Group** | **Species name** | **LDA_value** | **Group** | **Species name** | **LDA_value** | **Group** | **Species name** | **LDA_value** |
| --- | --- | --- | --- | --- | --- | --- | --- | --- |
| S-GP | unclassified_Actinomycetes | 3.5750 | S-LL | unclassified_Gaiellales | 3.1791 | S-SJ | Piscinibacter | 3.0849 |
|  | Sphingomicrobium | 3.8592 |  | unclassified_Solirubrobacteraceae | 4.1219 |  | Rubrivivax | 3.0217 |
|  | unclassified_Sphingomonadales | 3.0938 |  | unclassified_Gaiellaceae | 3.9795 |  | Novosphingobium | 3.4616 |
|  | Rudaea | 3.5226 | S-RH | Nitrospira | 3.4017 |  | Lysobacter | 3.2083 |
|  | unclassified_Mycobacteriales | 3.1591 |  | unclassified_Vicinamibacterales | 4.3299 |  | unclassified_Ilumatobacteraceae | 3.4703 |
| S-HF | unclassified_Gemmatimonadaceae | 3.7648 |  | Sphingomonas | 4.1296 |  | unclassified_Hyphomicrobiaceae | 3.2669 |
|  | unclassified_Candidatus_Limnocylindria | 3.4918 |  | Ferruginibacter | 3.0587 |  | unclassified_Betaproteobacteria | 3.0955 |
|  | unclassified_Kofleriaceae | 3.1652 |  | Rhizobium | 3.5072 |  | unclassified_Burkholderiaceae | 3.7914 |
|  | unclassified_Pyrinomonadaceae | 4.0869 |  | unclassified_Chloroflexota | 3.8259 |  | unclassified_Xanthobacteraceae | 3.2117 |
|  | unclassified_Dongiaceae | 3.3376 |  | Croceibacterium | 3.0266 | S-PS | Microvirga | 3.6362 |
|  | unclassified_Candidatus_Eiseniibacteriota | 3.0162 |  | unclassified_Chitinophagaceae | 3.8516 |  | Rubrobacter | 3.7480 |
|  | unclassified_Acidobacteriota | 4.0956 |  | Gaiella | 3.8061 |  | Pseudolysinimonas | 3.3393 |
|  | unclassified_Candidatus_Rokuibacteriota | 3.4724 |  | unclassified_Candidatus_Binatia | 3.2604 |  | unclassified_Actinomycetota | 4.0936 |
|  | unclassified_Burkholderiales | 3.7468 | S-SJ | unclassified_Pseudomonadota | 3.5427 |  | unclassified_Solirubrobacterales | 3.9313 |
|  | Candidatus_Acidiferrum | 3.1780 |  | Aquincola | 3.1890 |  | Marmoricola | 3.4668 |
|  | unclassified_Candidatus_Methylomirabilota | 3.7697 |  | Pseudomonas | 3.2842 |  | unclassified_Acidimicrobiales | 3.1389 |
|  | unclassified_d__Bacteria | 3.1056 |  | unclassified_Steroidobacteraceae | 3.9417 |  | unclassified_Thermoleophilaceae | 3.9047 |
|  | unclassified_Bacteroidota | 3.0676 |  | Phenylobacterium | 4.1787 |  | Microlunatus | 3.4533 |
|  | Pantoea | 3.8163 |  | unclassified_Alphaproteobacteria | 3.3490 |  | Agromyces | 3.4844 |
|  | unclassified_Myxococcota | 3.0151 |  | Variovorax | 3.7829 |  | unclassified_Kineosporiaceae | 3.1319 |
| S-HE | Sphingobium | 3.8746 |  | Pseudolabrys | 3.0565 |  | unclassified_Propionibacteriaceae | 4.1145 |
|  | Arthrobacter | 4.0171 |  | Ramlibacter | 3.1113 |  | Solirubrobacter | 3.4273 |
|  | Phycicoccus | 3.5552 |  | Luteibacter | 3.2340 |  | Pseudonocardia | 3.7842 |
|  | Streptomyces | 3.4383 |  | Rhizobacter | 3.3268 |  | Nakamurella | 3.3065 |
|  | Mesorhizobium | 3.3443 |  | unclassified_Micropepsaceae | 3.2216 |  | Mycobacterium | 3.3740 |
|  | unclassified_Casimicrobiaceae | 3.3693 |  | Reyranella | 3.2180 |  | Skermanella | 3.0331 |
|  | Pseudarthrobacter | 3.3843 |  | unclassified_Beijerinckiaceae | 3.1536 |  | unclassified_Rubrobacteraceae | 3.6547 |
|  | Microbacterium | 3.4704 |  | unclassified_Caulobacteraceae | 3.2237 |  | unclassified_Pseudonocardiales | 3.1090 |
| S-LL | Actinoplanes | 3.1311 |  | unclassified_Acidimicrobiia | 3.2767 |  | Nocardioides | 4.3631 |
|  | unclassified_Gemmatimonadales | 3.6788 |  | Povalibacter | 3.9010 |  | unclassified_Nocardioidaceae | 3.8774 |
|  | Lapillicoccus | 3.6369 |  | Usitatibacter | 3.4727 |  | Bradyrhizobium | 3.7647 |
|  | Ornithinibacter | 3.3159 |  | Hyphomicrobium | 3.1942 |  | unclassified_Candidatus_Limnocylindrales | 3.8604 |
|  |  |  |  |  |  |  | Blastococcus | 3.6041 |

**Table S10** LDA Values of Carbon-fixed Fungi

| **Group** | **Species name** | **LDA_value** |
| --- | --- | --- |
| S-PS | Rhynchospora | 5.3361 |

**Table S11** Correlation between Metabolic Microorganisms and Environmental Factors

| **Name** | **pH** | **MOAC** | **SOC** | **MBC** | **DOC** | **POC** | **TP** | **TN** | **TK** |
| --- | --- | --- | --- | --- | --- | --- | --- | --- | --- |
| unclassified_Vicinamibacterales | 0.1478 | -0.4034 | -0.4456 | -0.6358 | -0.5014 | -0.4680 | -0.1223 | -0.2028 | -0.4392 |
| unclassified_Actinomycetota | 0.0359 | 0.1352 | 0.1455 | -0.2077 | -0.0941 | -0.1286 | 0.7507 | -0.0231 | 0.3633 |
| Nocardioides | -0.1111 | 0.0969 | 0.1354 | 0.4144 | 0.2928 | 0.2490 | 0.1571 | 0.4227 | 0.5769 |
| unclassified_Solirubrobacteraceae | -0.2227 | 0.0887 | 0.1266 | 0.1185 | 0.1210 | 0.0980 | 0.5794 | 0.4502 | 0.5533 |
| unclassified_Gaiellaceae | -0.2063 | -0.0170 | 0.0192 | 0.0331 | 0.0383 | -0.0055 | 0.5313 | 0.5630 | 0.5744 |
| unclassified_Propionibacteriaceae | -0.4276 | 0.1264 | 0.1924 | 0.5291 | 0.4910 | 0.4740 | 0.1885 | 0.4697 | 0.5703 |
| unclassified_Candidatus_Limnocylindrales | 0.0998 | 0.0722 | 0.0737 | 0.1005 | 0.2950 | 0.2945 | -0.1504 | -0.5738 | 0.0408 |
| unclassified_Thermoleophilaceae | -0.3316 | 0.0925 | 0.1291 | 0.0813 | 0.1489 | 0.1237 | 0.6234 | 0.4516 | 0.5955 |
| Bradyrhizobium | -0.5178 | 0.2655 | 0.3305 | 0.7229 | 0.7854 | 0.7734 | -0.1054 | 0.0630 | 0.2431 |
| Arthrobacter | 0.1659 | -0.1489 | -0.1579 | -0.3096 | -0.4351 | -0.4488 | 0.1009 | 0.5922 | 0.2538 |
| Gaiella | 0.0801 | -0.1106 | -0.1000 | -0.0049 | -0.0192 | -0.0427 | 0.0520 | 0.4890 | 0.5955 |
| Sphingomonas | 0.1939 | -0.3224 | -0.3620 | -0.5378 | -0.4735 | -0.4707 | -0.2249 | 0.3390 | 0.1265 |
| unclassified_Acidobacteriota | 0.2060 | -0.1932 | -0.2376 | -0.4251 | -0.3328 | -0.3021 | -0.1610 | -0.4678 | -0.6275 |
| Sphingomicrobium | -0.2375 | -0.0854 | -0.0844 | 0.0394 | 0.1242 | 0.1527 | -0.0584 | 0.2314 | -0.0424 |
| unclassified_Gemmatimonadales | 0.0738 | -0.0805 | -0.0956 | -0.4360 | -0.4620 | -0.4795 | 0.5183 | 0.4675 | 0.2166 |
| unclassified_Nocardioidaceae | -0.2304 | 0.0640 | 0.1061 | 0.3227 | 0.2157 | 0.1735 | 0.3945 | 0.4615 | 0.5164 |
| unclassified_Steroidobacteraceae | -0.1607 | -0.2605 | -0.2565 | -0.1284 | -0.0805 | -0.0093 | -0.2202 | -0.1910 | -0.6001 |
| unclassified_Pyrinomonadaceae | 0.2367 | -0.2096 | -0.2598 | -0.8501 | -0.7975 | -0.7444 | 0.3075 | 0.0110 | -0.1336 |
| unclassified_Chloroflexota | 0.2943 | -0.2332 | -0.2850 | -0.5337 | -0.3158 | -0.3284 | -0.2263 | -0.3993 | -0.1823 |
| unclassified_Solirubrobacterales | 0.0398 | 0.3191 | 0.3426 | 0.2718 | 0.2693 | 0.2370 | 0.3400 | 0.2122 | 0.6513 |
| Pseudonocardia | -0.5521 | 0.1461 | 0.2091 | 0.4683 | 0.6136 | 0.5818 | 0.2455 | 0.3324 | 0.5194 |
| unclassified_Actinomycetes | -0.3766 | 0.0361 | 0.0710 | -0.1713 | 0.0493 | 0.0547 | 0.6537 | 0.3787 | 0.6360 |
| Variovorax | -0.1374 | -0.1987 | -0.1984 | 0.1700 | -0.0126 | 0.0038 | -0.5380 | 0.1425 | -0.4720 |
| unclassified_Xanthobacteraceae | -0.4759 | 0.0131 | 0.0756 | 0.7289 | 0.6190 | 0.5917 | -0.3509 | 0.1648 | -0.1634 |
| unclassified_Nitrososphaeraceae | 0.4120 | -0.0290 | -0.0797 | -0.5649 | -0.6147 | -0.5911 | 0.2633 | -0.1880 | -0.0348 |
| unclassified_Candidatus_Methylomirabilota | 0.3870 | -0.1472 | -0.2102 | -0.7095 | -0.5342 | -0.5068 | -0.0234 | -0.3357 | -0.1665 |
| Blastococcus | -0.3376 | 0.2063 | 0.2691 | 0.6870 | 0.6097 | 0.5501 | 0.0848 | 0.2529 | 0.3343 |
| Agromyces | 0.0952 | 0.2255 | 0.2540 | 0.3334 | 0.3213 | 0.3262 | -0.0837 | 0.3162 | 0.6795 |
| unclassified_Anaerolineales | 0.0557 | 0.3383 | 0.3247 | -0.2581 | -0.1024 | -0.0591 | 0.2358 | -0.3765 | -0.0539 |
| unclassified_Alphaproteobacteria | -0.5167 | -0.0066 | 0.0485 | 0.7100 | 0.7258 | 0.6672 | -0.1207 | -0.0619 | -0.1125 |
| unclassified_Burkholderiales | 0.3146 | -0.1571 | -0.2094 | -0.3621 | -0.2999 | -0.2720 | -0.2185 | -0.5435 | -0.6669 |
| Microvirga | -0.4509 | 0.2600 | 0.3130 | 0.8515 | 0.8894 | 0.8276 | -0.2811 | 0.0162 | 0.2042 |
| unclassified_Gemmatimonadaceae | 0.4024 | -0.3426 | -0.3944 | -0.8376 | -0.8664 | -0.8199 | 0.2199 | 0.0473 | -0.2322 |
| unclassified_Burkholderiaceae | 0.1566 | -0.0848 | -0.1192 | -0.1007 | -0.0640 | -0.0564 | -0.5414 | -0.3957 | -0.6010 |
| Solirubrobacter | -0.4468 | 0.1604 | 0.2129 | 0.4243 | 0.4412 | 0.3979 | 0.2972 | 0.4246 | 0.4567 |
| unclassified_Rubrobacteraceae | -0.4336 | 0.1434 | 0.1927 | 0.4286 | 0.4226 | 0.3848 | 0.3553 | 0.4241 | 0.5966 |
| Rubrobacter | -0.3996 | 0.1620 | 0.2168 | 0.4440 | 0.3826 | 0.3640 | 0.4268 | 0.3850 | 0.4345 |
| Lapillicoccus | -0.3154 | 0.0296 | 0.0806 | 0.3679 | 0.2217 | 0.2135 | 0.1779 | 0.6706 | 0.3915 |
| unclassified_Acidimicrobiia | -0.3417 | 0.0493 | 0.0984 | 0.6041 | 0.4658 | 0.4631 | -0.2119 | -0.0600 | -0.3603 |
| unclassified_Candidatus_Binatia | 0.3821 | 0.0088 | -0.0504 | -0.4815 | -0.4937 | -0.5315 | 0.3270 | -0.3016 | 0.0257 |
| Rhizobium | 0.2847 | -0.2069 | -0.2335 | 0.0137 | -0.1478 | -0.1823 | -0.5480 | -0.0542 | -0.3129 |
| unclassified_Ilumatobacteraceae | 0.0675 | 0.1149 | 0.1367 | 0.5129 | 0.3892 | 0.3799 | -0.3181 | -0.4785 | -0.4296 |
| Phenylobacterium | -0.3236 | -0.1385 | -0.1203 | 0.2786 | 0.1494 | 0.1648 | -0.2750 | 0.0812 | -0.5859 |
| Marmoricola | -0.1659 | 0.0881 | 0.1244 | 0.2910 | 0.1861 | 0.1713 | 0.2853 | 0.5110 | 0.5867 |
| Microbacterium | 0.1245 | 0.1735 | 0.2135 | 0.6090 | 0.4899 | 0.4745 | -0.3451 | 0.1015 | 0.3797 |
| Mesorhizobium | -0.1081 | 0.0317 | 0.0280 | 0.0148 | -0.0487 | -0.0870 | 0.1037 | 0.2072 | -0.1270 |
| unclassified_Dongiaceae | -0.1451 | -0.1631 | -0.1718 | -0.2989 | -0.1845 | -0.1352 | -0.0236 | -0.2658 | -0.5476 |
| Streptomyces | 0.0425 | -0.0772 | -0.0671 | 0.2849 | 0.0651 | 0.0022 | -0.1143 | 0.3910 | 0.2360 |
| unclassified_Acidimicrobiales | -0.4605 | -0.0290 | 0.0354 | 0.5606 | 0.5900 | 0.5829 | 0.1023 | 0.0581 | 0.1142 |
| Nitrospira | 0.0381 | -0.0569 | -0.0693 | -0.3271 | -0.1576 | -0.1352 | -0.0706 | 0.2290 | 0.6174 |

**Table S12** Correlation between Fixed Microorganisms and Environmental Factors

| **Name** | **pH** | **MBC** | **DOC** | **POC** | **TN** | **TK** | **TP** | **MOAC** | **SOC** |
| --- | --- | --- | --- | --- | --- | --- | --- | --- | --- |
| unclassified_Vicinamibacterales | 0.1654 | -0.5740 | -0.5189 | -0.4844 | -0.2380 | -0.5678 | -0.1265 | -0.3503 | -0.3913 |
| unclassified_Actinomycetota | 0.0521 | -0.2556 | -0.1122 | -0.1533 | 0.0377 | 0.4252 | 0.6584 | 0.0996 | 0.1069 |
| Nocardioides | -0.0773 | 0.4125 | 0.2885 | 0.2321 | 0.3800 | 0.5495 | 0.1688 | 0.1396 | 0.1768 |
| unclassified_Gaiellaceae | -0.1980 | 0.0799 | 0.0317 | 0.0115 | 0.6068 | 0.6059 | 0.4493 | -0.0673 | -0.0277 |
| unclassified_Solirubrobacteraceae | -0.2649 | 0.1330 | 0.1128 | 0.0991 | 0.5096 | 0.5103 | 0.5500 | 0.0974 | 0.1395 |
| unclassified_Propionibacteriaceae | -0.4172 | 0.5214 | 0.4932 | 0.4631 | 0.4763 | 0.5747 | 0.1924 | 0.1368 | 0.2020 |
| unclassified_Nitrososphaeraceae | 0.4386 | -0.5679 | -0.6289 | -0.6004 | -0.2053 | -0.0493 | 0.2783 | -0.0022 | -0.0515 |
| unclassified_Candidatus_Limnocylindrales | 0.1525 | -0.0605 | 0.1604 | 0.1390 | -0.4524 | 0.1566 | -0.0881 | -0.0066 | -0.0195 |
| Bradyrhizobium | -0.5444 | 0.7782 | 0.7750 | 0.7783 | 0.1170 | 0.2045 | -0.1966 | 0.1959 | 0.2683 |
| unclassified_Acidobacteriota | 0.1574 | -0.4448 | -0.3344 | -0.2994 | -0.4397 | -0.5985 | -0.1371 | -0.2162 | -0.2535 |
| Gaiella | 0.0584 | -0.0441 | -0.0175 | -0.0712 | 0.4708 | 0.6790 | 0.0801 | -0.0952 | -0.0825 |
| unclassified_Thermoleophilaceae | -0.3108 | 0.1081 | 0.1905 | 0.1489 | 0.3709 | 0.5826 | 0.6156 | 0.1182 | 0.1532 |
| Sphingomonas | 0.2019 | -0.4954 | -0.4576 | -0.4483 | 0.3321 | 0.1095 | -0.1994 | -0.3010 | -0.3417 |
| unclassified_Nocardioidaceae | -0.2874 | 0.2770 | 0.2124 | 0.1752 | 0.4598 | 0.4756 | 0.4624 | 0.0060 | 0.0438 |
| unclassified_Gemmatimonadales | -0.0217 | -0.4954 | -0.4346 | -0.4795 | 0.4576 | 0.2765 | 0.6462 | -0.0848 | -0.0970 |
| unclassified_Chloroflexota | 0.3516 | -0.4440 | -0.2906 | -0.2950 | -0.5440 | -0.3296 | -0.2575 | -0.1549 | -0.2181 |
| unclassified_Pyrinomonadaceae | 0.2526 | -0.8507 | -0.7898 | -0.7477 | 0.0129 | -0.1457 | 0.3387 | -0.1505 | -0.1995 |
| Arthrobacter | 0.0845 | -0.2441 | -0.3290 | -0.3514 | 0.6508 | 0.4419 | 0.1023 | -0.1489 | -0.1450 |
| unclassified_Steroidobacteraceae | -0.0436 | -0.0517 | -0.1303 | -0.0903 | -0.2380 | -0.7472 | -0.2124 | -0.1987 | -0.1976 |
| unclassified_Solirubrobacterales | 0.0121 | 0.2762 | 0.3191 | 0.2742 | 0.1668 | 0.6516 | 0.3312 | 0.3766 | 0.3982 |
| Pseudonocardia | -0.5315 | 0.4347 | 0.5911 | 0.5796 | 0.3206 | 0.5812 | 0.2881 | 0.1998 | 0.2587 |
| Sphingomicrobium | -0.2268 | 0.0096 | 0.0832 | 0.1215 | 0.3264 | 0.0665 | -0.0481 | -0.0525 | -0.0493 |
| unclassified_Actinomycetes | -0.3006 | -0.1596 | 0.0564 | 0.0509 | 0.3187 | 0.6152 | 0.6528 | 0.0974 | 0.1310 |
| unclassified_Xanthobacteraceae | -0.5724 | 0.5907 | 0.6097 | 0.6223 | 0.2031 | -0.1013 | -0.4226 | -0.1713 | -0.1115 |
| Variovorax | -0.1229 | 0.1533 | -0.0137 | 0.0016 | 0.1403 | -0.4482 | -0.5450 | -0.2786 | -0.2771 |
| unclassified_Acidimicrobiia | -0.1459 | 0.6687 | 0.5583 | 0.5479 | -0.1913 | -0.2327 | -0.2324 | 0.2781 | 0.3039 |
| Blastococcus | -0.3431 | 0.6495 | 0.5534 | 0.5194 | 0.3173 | 0.3874 | 0.1321 | 0.1741 | 0.2379 |
| unclassified_Anaerolineales | 0.1042 | -0.3419 | -0.1828 | -0.1144 | -0.3187 | -0.0542 | 0.2155 | 0.2365 | 0.2157 |
| unclassified_Gemmatimonadaceae | 0.2504 | -0.8542 | -0.8927 | -0.8451 | 0.1241 | -0.2686 | 0.3773 | -0.3432 | -0.3796 |
| unclassified_Burkholderiales | 0.3110 | -0.4212 | -0.3525 | -0.3339 | -0.5220 | -0.6385 | -0.1902 | -0.1494 | -0.2001 |
| unclassified_Candidatus_Methylomirabilota | 0.3875 | -0.7360 | -0.5380 | -0.5249 | -0.3024 | -0.1511 | -0.0072 | -0.1790 | -0.2360 |
| unclassified_Burkholderiaceae | 0.1895 | -0.0591 | -0.0208 | -0.0389 | -0.3473 | -0.6007 | -0.5825 | -0.1396 | -0.1727 |
| unclassified_Acidimicrobiales | -0.2597 | 0.4609 | 0.4609 | 0.4428 | -0.0545 | 0.1284 | 0.0106 | -0.0591 | -0.0077 |
| unclassified_Alphaproteobacteria | -0.5197 | 0.6547 | 0.5900 | 0.5769 | 0.1907 | -0.1506 | -0.3684 | -0.0586 | 0.0005 |
| Agromyces | 0.0014 | 0.4270 | 0.4390 | 0.4182 | 0.2367 | 0.6316 | -0.1051 | 0.1888 | 0.2154 |
| Streptomyces | 0.1188 | 0.1393 | -0.0531 | -0.1024 | 0.3781 | 0.2979 | -0.0234 | -0.1434 | -0.1318 |
| Microvirga | -0.4509 | 0.8625 | 0.8856 | 0.8758 | 0.0327 | 0.1823 | -0.2906 | 0.2124 | 0.2768 |
| Solirubrobacter | -0.3845 | 0.4869 | 0.4652 | 0.4822 | 0.3299 | 0.3576 | 0.2680 | 0.1626 | 0.2225 |
| Rubrobacter | -0.2951 | 0.3542 | 0.3054 | 0.2759 | 0.3167 | 0.4884 | 0.5149 | 0.2271 | 0.2751 |
| Lapillicoccus | -0.3601 | 0.3446 | 0.2189 | 0.2146 | 0.7072 | 0.4156 | 0.1880 | 0.0487 | 0.0992 |
| Nitrospira | 0.1103 | -0.3632 | -0.1845 | -0.1680 | 0.2416 | 0.5703 | -0.1501 | 0.0142 | -0.0074 |
| unclassified_Candidatus_Binatia | 0.3560 | -0.4341 | -0.4209 | -0.4444 | -0.3046 | 0.0468 | 0.2007 | 0.0268 | -0.0140 |
| Marmoricola | -0.2274 | 0.3085 | 0.1987 | 0.1697 | 0.5407 | 0.5911 | 0.2939 | 0.0203 | 0.0671 |
| Phenylobacterium | -0.1807 | 0.2693 | 0.1073 | 0.1193 | -0.0649 | -0.6606 | -0.2244 | -0.1089 | -0.0995 |
| unclassified_Rubrobacteraceae | -0.3747 | 0.3744 | 0.3766 | 0.3295 | 0.3946 | 0.5714 | 0.4479 | 0.1648 | 0.2201 |
| unclassified_Candidatus_Limnocylindria | 0.6371 | -0.8099 | -0.7313 | -0.7323 | -0.0795 | 0.1098 | 0.2049 | -0.1062 | -0.1606 |
| unclassified_Ilumatobacteraceae | 0.1676 | 0.3172 | 0.2271 | 0.2452 | -0.5806 | -0.4652 | -0.4652 | 0.1128 | 0.1104 |
| Rhizobium | 0.2178 | 0.0654 | -0.1242 | -0.1554 | 0.0096 | -0.3806 | -0.5447 | -0.2326 | -0.2499 |
| unclassified_Chitinophagaceae | 0.2987 | -0.6123 | -0.6010 | -0.5375 | -0.2389 | -0.2522 | 0.0311 | -0.1018 | -0.1496 |
| unclassified_Pseudomonadota | -0.3258 | 0.0991 | 0.2501 | 0.2720 | 0.0707 | -0.2568 | -0.4173 | -0.1357 | -0.1283 |

**Table S13 Combined abbreviations and their corresponding full meanings**

| **Abbrevlations** | **Meaning of representation** |
| --- | --- |
| P-HE | Plant of *Hosta ensata* F. Maek. |
| S-HE | Rhizosphere microorganism of *Hosta ensata* F. Maek. |
| RS-HE | Rhizosphere soil of *Hosta ensata* F. Maek. |
| P-PS | Plant of *Pseudolysimachion spicatum* (L.) Opiz |
| S-PS | Rhizosphere microorganism of *Pseudolysimachion spicatum* (L.) Opiz |
| RS-PS | Rhizosphere soil of *Pseudolysimachion spicatum* (L.) Opiz |
| P-RH | Plant of *Rudbeckia hirta L*. |
| S-RH | Rhizosphere microorganism of *Rudbeckia hirta* L. |
| RS-RH | Rhizosphere soil of *Rudbeckia hirta* L. |
| P-GP | Plant of *Gaillardia pulchella* Foug. |
| S-GP | Rhizosphere microorganism of *Gaillardia pulchella* Foug. |
| RS-GP | Rhizosphere soil of *Gaillardia pulchella* Foug. |
| P-SJ | Plant of *Salvia japonica*Thunb. |
| S-SJ | Rhizosphere microorganism of *Salvia japonica*Thunb. |
| RS-SJ | Rhizosphere soil of *Salvia japonica*Thunb. |
| P-LL | Plant of *Lycopus lucidus*Turcz. ex Benth. |
| S-LL | Rhizosphere microorganism of *Lycopus lucidus*Turcz. ex Benth. |
| RS-LL | Rhizosphere soil of *Lycopus lucidus*Turcz. ex Benth. |
| P-HF | Plant of *Hemerocallis* *fulva*‘Golden Doll’ |
| S-HF | Rhizosphere microorganism of *Hemerocallis fulva*‘Golden Doll’ |
| RS-HF | Rhizosphere soil of *Hemerocallis fulva*‘Golden Doll’ |

**Attached figure**


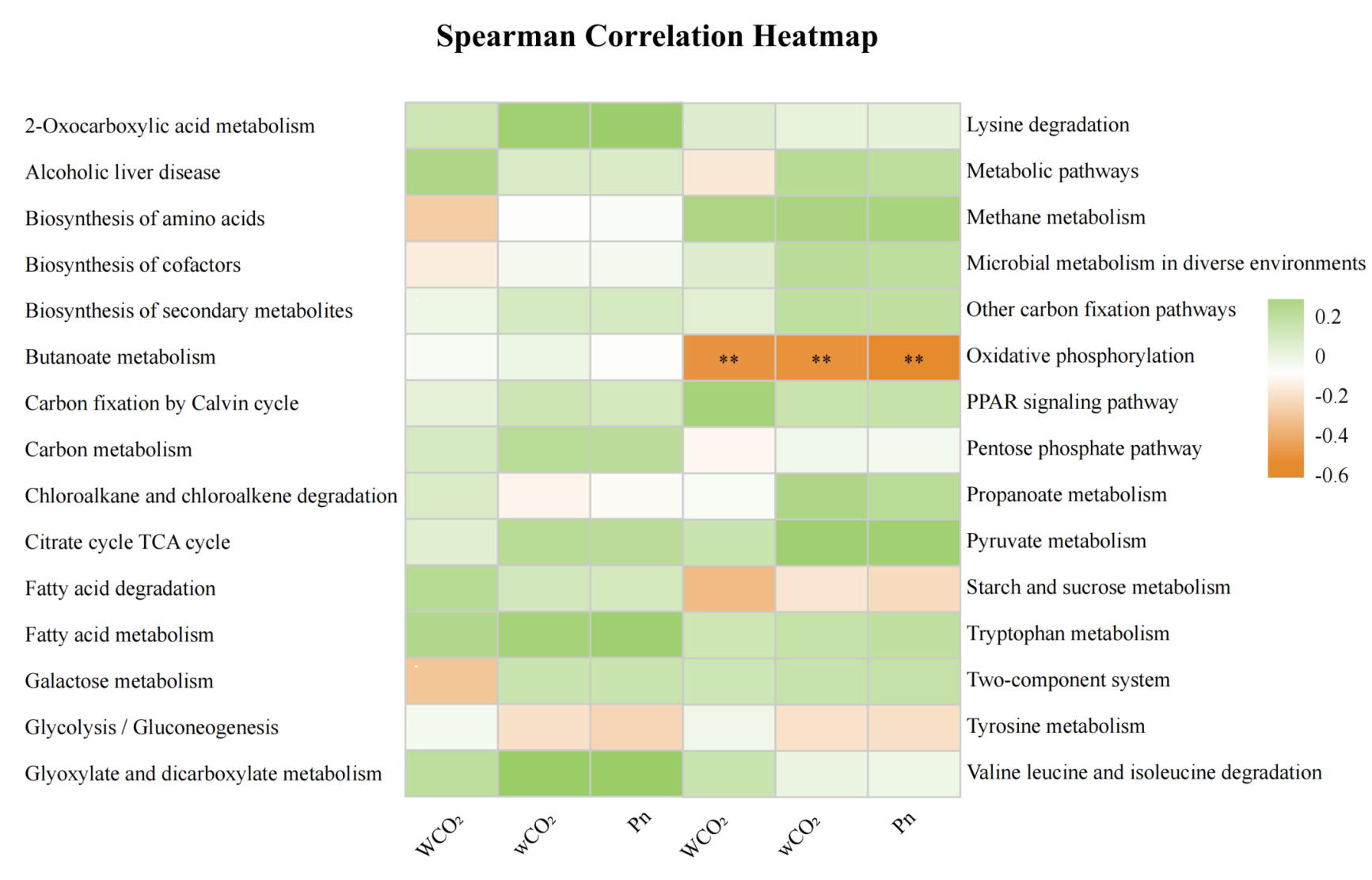


**Figure S1** Correlation heatmap of Pn, WCO₂, wCO₂, and metabolic pathways
